# Exploring the Therapeutic Potential of *Bupleurum* in Medical Treatment: A Comprehensive Overview

**DOI:** 10.3390/ph18091331

**Published:** 2025-09-05

**Authors:** Yu Tian, Jiageng Guo, Xinya Jiang, Hongyu Lu, Jinling Xie, Fan Zhang, Zhengcai Du, Erwei Hao

**Affiliations:** 1 Guangxi Key Laboratory of Efficacy Study on Chinese Materia Medica, Guangxi University of Chinese Medicine, Nanning 530000, China; 2 University Engineering Research Center of Reutilization of Traditional Chinese Medicine Resources, Nanning 530000, China; 3 Guangxi Key Laboratory of TCM Formulas Theory and Transformation for Damp Diseases, Guangxi University of Chinese Medicine, Nanning 530000, China; 4 Institute of Traditional Chinese and Zhuang-Yao Ethnic Medicine, Guangxi University of Chinese Medicine, Nanning 530000, China; 5 Engineering Research Center of Innovative Drugs for Tradtional Chinese Medicine and Zhuang & Yao Medicine, Ministry of Education, Guangxi University of Chinese Medicine, Nanning 530000, China

**Keywords:** Chinese medicine, *Bupleurum*, pharmacological effects, mechanism of action, research progress

## Abstract

*Bupleurum* is a Chinese medicinal material widely used in clinical practice. Its medicinal component is the dried roots of either the Umbrella plant *Bupleurum chinense* DC or *Bupleurum scorzonerifolium* Willd. This review systematically searched major scientific databases such as Web of Science, PubMed, and ScienceDirect, and found that it contains various bioactive substances including saikosaponins, polysaccharides, flavonoids, and volatile oils. These components have demonstrated significant efficacy in anti-tumor, anti-inflammatory, and neuroprotective activities. Research has confirmed that this medicinal herb can exert its pharmacological effects by promoting tumor cell apoptosis, inhibiting cell proliferation, regulating inflammatory signaling pathways, and alleviating neuroinflammation. Additionally, its antipyretic and antiviral properties have also garnered widespread attention. However, clinical data regarding its optimal dosage, administration routes, and safety are still insufficient, necessitating further trials for validation. Investigating the synergistic effects of *Bupleurum* with other drugs and the safety of its use in different populations are also key directions of current research. Given the urgent need for efficient and sustainable healthcare in modern society, a deep understanding of the mechanisms and safety of *Bupleurum* is of significant importance for its validation as a foundation for new drug development. In summary, *Bupleurum*, as a multifunctional natural product, has broad application prospects and is expected to play a greater role in future medical research and clinical practice.

## 1. Introduction

**Figure 1:**
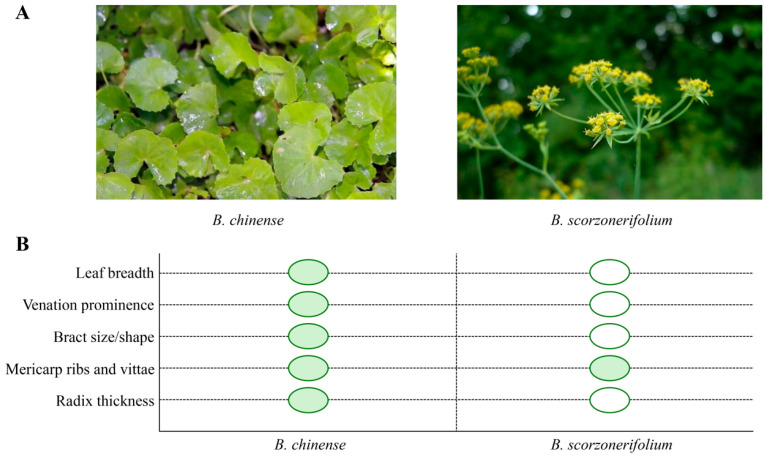
Diagnostic comparison of *Bupleurum* species. Panel (**A**) presents representative photos of *B. chinense* and *B. scorzonerifolium*. Panel (**B**) summarizes the same traits across the two species in a qualitative, dot-matrix format; filled circles indicate traits that are typically prominent for the species, whereas open circles indicate traits that are present but less prominent. Image credits: Left panel (*B. chinense*)—Wikimedia Commons, CC BY-SA 4.0. Right panel (*B. scorzonerifolium*)—Wikimedia Commons, CC BY-SA 4.0. Changes: cropped for layout; no other edits.

*Bupleurum* is a Chinese medicinal herb widely used in clinical practice. Its medicinal component consists of the dried roots of either *Bupleurum chinense* DC or *Bupleurum scorzonerifolium* Willd. This herb is habitually called Gu Cao, Shan Cai, or Chai Cao in folk medicine [1]. Within the realm of traditional medicine, *Bupleurum* is extensively utilized to address various symptoms, including fever, chills, irregular menstruation, and qi deficiency [2]. This review encompasses research findings on two species: *Bupleurum chinense* DC and *Bupleurum scorzonerifolium* Willd. To improve botanical clarity, Figure 1 illustrates representative *Bupleurum* material with key diagnostic traits highlighted. In accordance with floristic treatments, the genus is recognized by simple, entire leaves and conspicuous bracts/bracteoles. For the two pharmacopeial sources—*B. chinense* DC and *B. scorzonerifolium* Willd—the traits most commonly emphasized include leaf outline and venation, size/shape of involucre and involucel bracts in the compound umbel, and mericarp morphology (ribs and vittae). These visual references support voucher-based identification and harmonize the plant material across studies. Due to the diverse sources of medicinal *Bupleurum* and the variations in specific materials used across different studies, this article does not establish a fixed species ratio. The quality control of *Bupleurum* medicinal materials in the Chinese Pharmacopoeia is primarily based on the content of its signature active components, saikosaponin A and saikosaponin D, rather than strictly limiting the species ratio. In recent years, significant progress has been made in research into *Bupleurum* across multiple aspects. On the one hand, through the analysis of chemical components, in-depth exploration has been conducted on the extraction and identification of active substances such as flavonoids and saponins. On the other hand, research focusing on single pharmacological mechanisms, such as anti-cancer [3,4], anti-inflammatory [5], anti-depressant [6,7], and neuroprotective effects [8], has laid a solid theoretical foundation for the clinical application of *Bupleurum*. However, existing reviews are mostly confined to specific disease areas or single component analyses, lacking a systematic integration of the overall therapeutic effects of *Bupleurum*. Unlike previous discussions focusing on single components or specific diseases, this review systematically elucidates the pharmacological effects and mechanisms of *Bupleurum* in multiple fields, including anti-tumor [9,10], anti-inflammatory [11,12], antiviral [13], and neuroprotective activities, through a comprehensive analysis of the existing literature, thereby fully revealing its significant value in disease treatment.

This review targets researchers in traditional Chinese medicine, pharmacologists, and developers of natural medicines, systematically integrating multidisciplinary research findings on *Bupleurum*. It focuses on analyzing its chemical components and multidimensional pharmacological activities such as anti-tumor, anti-inflammatory, and anti-depressant effects. The study aims to elucidate multi-target network pharmacology mechanisms, overcome the translational medicine bottlenecks in standardized formulation and clinical safety evaluation, and coordinate resource development with ecological sustainability. By constructing a comprehensive “basic research-clinical translation-industrial development” framework, it is grounded in the theories of classical Chinese medicine prescriptions while aligning with international standards. This provides a scientific basis for the development of *Bupleurum* resources and clinical applications, promoting the inheritance of traditional Chinese medicine culture and industrial upgrading.

## 2. Review Methods

To comprehensively review the pharmacological effects of various *Bupleurum* species, we conducted a systematic search across major scientific databases, including Medline, PubMed, ScienceDirect, and Scopus, covering the publication period from January 1, 2018 to June 30, 2025. Additionally, we performed a manual search to identify additional relevant articles that may have been overlooked in the database searches. The literature search process is intended to cover a wide range of studies discussing the molecular mechanisms and therapeutic potential of *Bupleurum* from multiple species of *Bupleurum*, including but not limited to *Bupleurum chinense* DC and *Bupleurum scorzonerifolium* Willd. The search strategy employs specific keywords, including “*Bupleurum*”, “*Bupleurum* saponin”, “*Bupleurum* polysaccharide”, “Main components of *Bupleurum*”, “Mechanism of action”, and “pharmacological action.” Additionally, relevant synonyms and related terms are incorporated. Boolean operators (AND/OR) are utilized to ensure comprehensive coverage of all pertinent studies concerning the pharmacological effects of *Bupleurum*. The inclusion criteria for this review are pre-defined to consider peer-reviewed research articles, reviewed papers, and clinical trial reports published in English. Studies featuring in vitro and in vivo experiments were included to provide a complete overview of the pharmacological characteristics of *Bupleurum*. Articles that are not directly related to the pharmacological effects of *Bupleurum* or only focus on its chemical synthesis without involving biological effects were excluded.

## 3. Chemical Composition

*Bupleurum*, a Chinese medicinal herb commonly used in clinical practice, contains a variety of active ingredients. Among these, saikosaponins constitute 1.5–3.5% and are the core active components, with saikosaponin accounting for 30–40% of the total saponins. The polysaccharide content ranges from 2% to 5%, with neutral polysaccharides making up approximately 70%. The flavonoid content is between 0.2% and 1.5%, volatile oils constitute 0.1–0.3%, coumarins range from 0.05% to 0.2%, and polyacetylenes are present at 0.01–0.1%. The specific levels of these components can vary depending on the variety, origin, and harvesting period of the *Bupleurum*. In Table 1, we present a table-style, class-organized overview of representative constituents of *Bupleurum*, including saponins, polysaccharides, flavonoids, volatile oils, coumarins, and polyacetylenes. The RG-I, RG-II, and several Chaihu polysaccharide grading samples (such as III5311, 2IIb, 2IIc) involved in this study are all natural polysaccharide/polysaccharide domains with slight heterogeneity in composition and substitution, and some are process grading names rather than single compounds. Therefore, a unique two-dimensional structural formula is not provided. For each compound, the figure provides the name, chemical class, standardized structural formula, and literature reference, offering a concise and accurate summary of the phytochemical landscape.

**Table 1:** Representative constituents of *Bupleurum* organized by chemical class. Listed are triterpenoid saponins (saikosaponin A, saikosaponin B1, saikosaponin B2), a pectic polysaccharide repeat unit (rhamnogalacturonan-I—RG-I; drawn as a schematic repeat with n indicating the degree of polymerization), the coumarin scopoletin, and the polyacetylene bupleurynol. All structures were prepared using a unified template (bond lengths/widths, fonts, and stereochemical notation) for consistency. The table highlights the characteristic components of Bupleurum and cites representative sources. Abbreviations: SSa—saikosaponin A; SSb1/SSb2—saikosaponin b1/b2; RG-I—rhamnogalacturonan-I.

Category	Chemical Name	Structural Formula	References
Saponins	Saikosaponin A	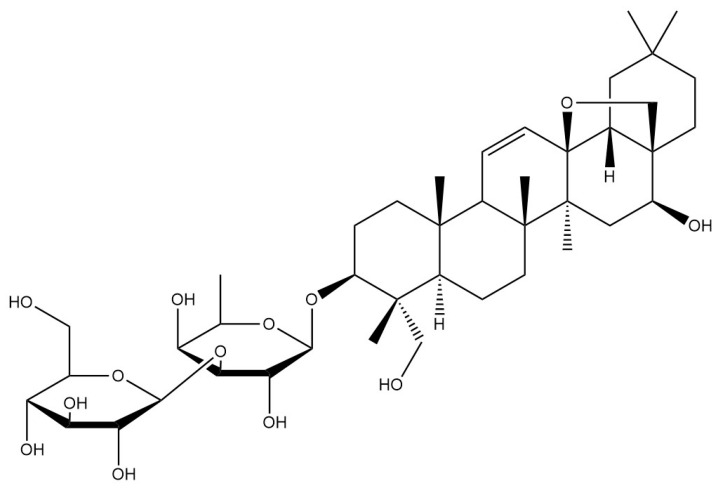	[14]
	Saikosaponin B1	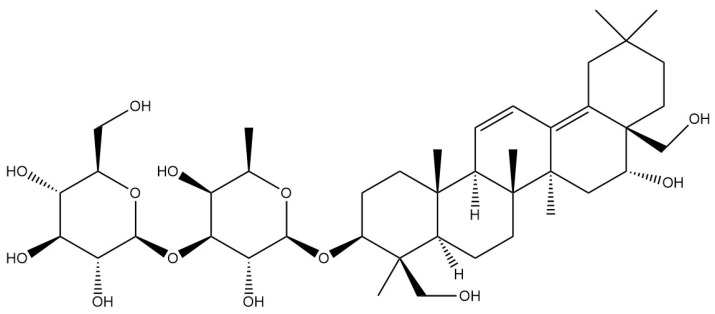	[14]
	Saikosaponin B2	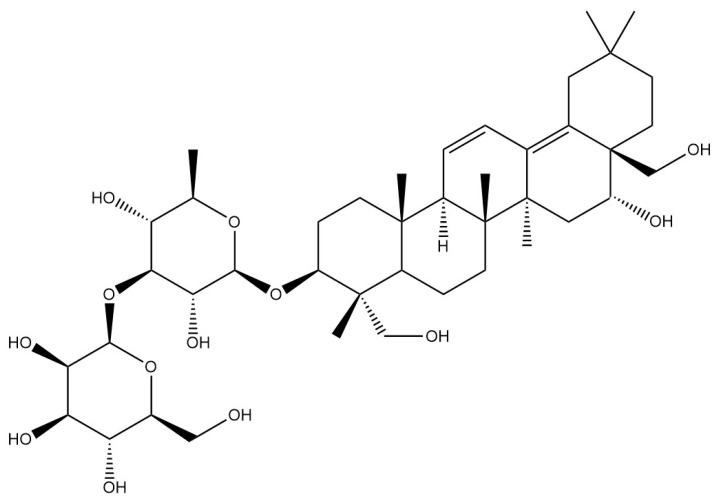	[14]
Polysaccharides	Type I rhamnogalacturonan	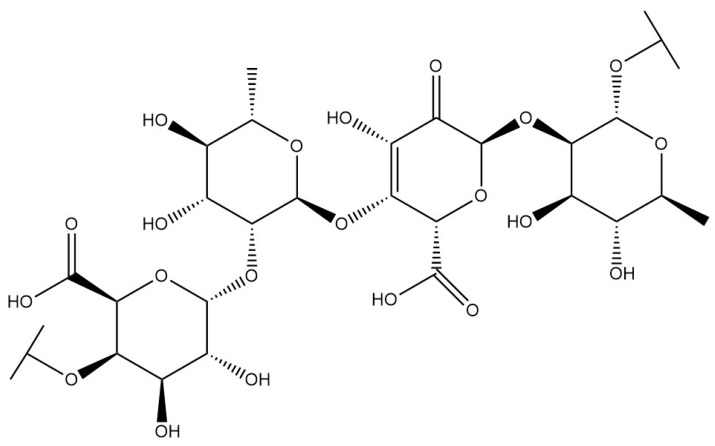	[15]
Coumarins	Scopoletin	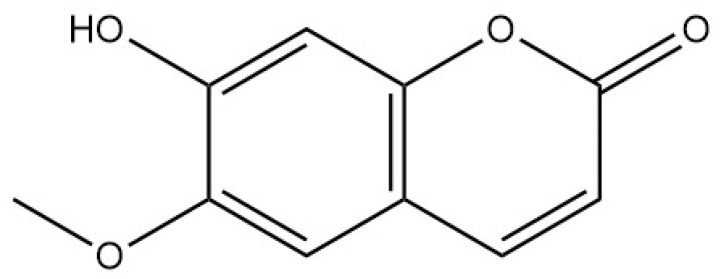	[16]
Polyacetylenes	Bupleurynol	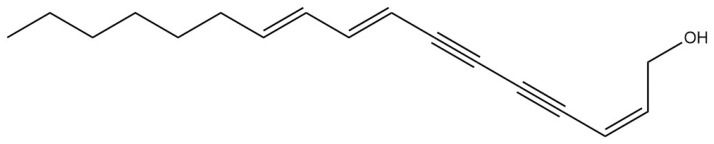	[17]

*Bupleurum* saponin is one of the active components of *Bupleurum* [18]. To date, more than a hundred different saikosaponins have been identified, among which saikosaponin A, saikosaponin B1, saikosaponin B2, and saikosaponin D are common and important saponin components [19].Their structure is formed by the linkage of a hydrophobic sapogenin and a hydrophilic sugar chain through a glycosidic bond. Due to the presence of carboxyl or uronic acid groups, saikosaponins exhibit acidic properties. Furthermore, they are soluble in water and possess foaming and hemolytic properties. Under acidic or alkaline conditions, their glycosidic bonds are susceptible to hydrolysis, resulting in the formation of sapogenin and sugar. All saikosaponins are pentacyclic triterpenoid oleanane-type derivatives. The sapogenin structures primarily include seven different types: epoxy ether, isocyclic diene, 12-ene, homocyclic diene, 12-ene-28-carboxylic acid, isocyclodiene-30-carboxylic acid, and 18-ene type [20]. *Bupleurum* saponin A and *Bupleurum* saponin D can be used as criteria for testing the quality of *Bupleurum*, among which *Bupleurum* saponin D is considered to be the ingredient with the strongest pharmacological activity.

*Bupleurum* polysaccharide is one of the main active components of *Bupleurum*. It has a complex composition, containing various monosaccharide and polysaccharide structures. Representative components include L-arabinose, ribose, D-xylose, L-rhamnose, D-glucose, and D-galacturonic acid, as well as type I rhamnogalacturonan (RG-I) and type II rhamnogalacturonan (RG-II). *Bupleurum* polysaccharide is one of the main active ingredients of *Bupleurum*. It is mainly composed of L-arabinosyl (arabinogalactan (AG)-type pectin), ribosyl, D-xylosyl, L-rhamnosyl, type I rhamnogalacturonan (RG-I), type II rhamnogalacturonan (RG-II), D-glucosyl (D-xylose, D-glucose), D-galacturonic acid (HG), and so on. In addition, there are also a small amount of *Bupleurum* polysaccharide III5311, *Bupleurum* polysaccharide 2IIb, and *Bupleurum* polysaccharide 2IIc [15,21]. *Bupleurum* polysaccharide III is primarily composed of seven monosaccharides, which include xylose, rhamnose, arabinose, mannose, galacturonic acid, galactose, and glucose. *Bupleurum* polysaccharide is readily soluble in water but difficult to dissolve in organic solvents, and it is prone to glycosidic bond hydrolysis under strong acid or strong alkali conditions. Its aqueous solution exhibits non-Newtonian fluid characteristics, maintaining stability through steric hindrance and electrostatic repulsion.

*Bupleurum* is rich in flavonoids. Fengchun Yang et al. [22] isolated and identified quercetin, isorhamnetin, isoblastin-3-O-glucoside, puerarin, and rutin from *Bupleurum*. Wang Ning et al. [14] further isolated two additional flavonoid compounds: kaempferol-3-O-α-L-arabinoside and kaempferol-3,7-di-O-α-L-rhamnoside. Woyuan et al. [23] isolated kaempferol-7-rhamnoside, kaempferol-3,7-di-O-rhamnoside, kaempferol-3-O-α-L-arabinofuranoside-7-O-α-L-rhamnopyranoside. Additionally, 7,4′-dihydroxy-isoflavone-7-O-β-D-glucoside has been isolated from the roots of *Bupleurum*. The flavonoid components of *Bupleurum* can undergo color reactions, such as turning red or purple when reacting with hydrochloric acid–magnesium powder. They are also capable of undergoing hydrolysis or oxidation reactions under both acidic and alkaline conditions. Furthermore, due to the presence of phenolic hydroxyl groups, the flavonoid components of *Bupleurum* demonstrate antioxidant properties.

By isolating and identifying the volatile oils in *Bupleurum*, studies have found that its main components are terpenoids and aliphatic compounds [24,25], including (-)-β-elemene and (-)-β-cadinene et al. Further separation found that the volatile oils also contained heptanal, α-pinene, 2-pentylfuran, dodecanal, (Z)-6-tridecen-4-yne, calarene, germacrene D, caryophyllene oxide, and α-caryophyllene alcohol [26]. The volatile oil of *Bupleurum* exhibits strong lipophilicity, volatility, and a susceptibility to oxidation when exposed to light. Therefore, protection from light, low temperatures, and an inert environment are essential for preserving the activity of its components.

Coumarins isolated from *Bupleurum* are generally simple coumarins, such as scopoletin, esculetin, fraxetin, limettin, and diesters of pyranocoumarins [16]. Xia Zhaodi et al. [15] found that there is angenomalin, a type of pyranocoumarin, in *Bupleurum*. Polyynes are also one of the effective active components of *Bupleurum*. Fang Yuan et al. [17] isolated and purified four polyyne compounds from *Bupleurum* using chromatographic techniques, including silica gel column chromatography, reverse phase chromatography, and preparative chromatography. These compounds were identified as (2Z,8Z,10E)-2,8,10-pentadecatrien-4,6-diyne-1-ol, (2Z,8E,10E)-2,8,10-pentadecatrien-4,6-diyne-1-ol, (2Z,8Z,10E)-2,8,10-heptadecatrien-4,6-diyne-1-ol, and bupleurynol. In addition to the aforementioned chemical components, there are also benzyl alcohol derivatives, lignans, organic acids (such as oleic acid, linolenic acid, palmitic acid, and stearic acid), and alcohols (such as α-spinasterol, adonitol, stigmasterol, and bupleurumol). Coumarin chemical components exhibit good solubility in organic solvents, but strong acidic conditions can trigger irreversible hydrolysis and ring opening, while strong alkali conditions or high temperatures may lead to oxidative degradation or polymerization. Reversible ring opening reactions can only occur in cold, dilute alkali environments. Polyacetylenic compounds, on the other hand, are highly lipophilic and prone to photocycloaddition or oxidative cleavage upon exposure to light, thus requiring strict protection from light.

The interaction of these chemical components has enabled *Bupleurum* to be widely used in the field of medicine for the treatment of a variety of diseases.

## 4. Pharmacological Effect

**Figure 2:**
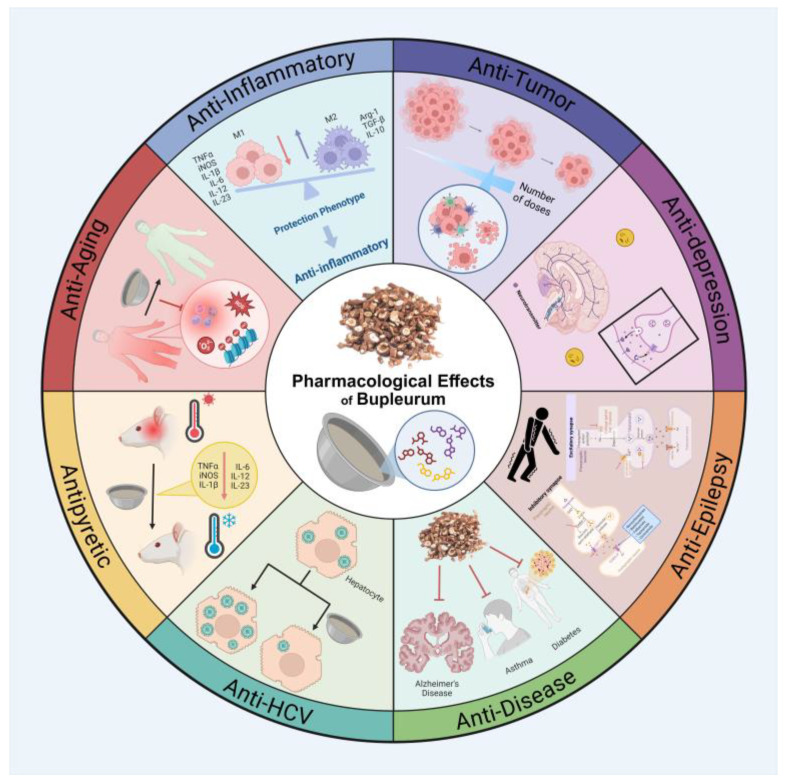
Pharmacological Effects of *Bupleurum*. Schematic overview summarizing the main pharmacological activities reported for *Bupleurum*, including anti-tumor, anti-inflammatory, anti-viral, neuroprotective, anti-pyretic and metabolic modulation effects. Arrows indicate the direction of regulation (inhibition or activation) on representative pathways (e.g., NF-κB, PI3K/AKT/mTOR, STAT3, SIRT1/Nrf2). Boxes list exemplar constituents (saikosaponins, polysaccharides, flavonoids, volatile oils, coumarins, and polyacetylenes). Abbreviations: NF-κB—nuclear factor-κB; PI3K—phosphatidylinositol-3-kinase; AKT—protein kinase B; mTOR—mechanistic target of rapamycin; STAT3—signal transducer and activator of transcription 3; SIRT1—sirtuin-1; Nrf2—nuclear factor erythroid 2–related factor 2.

As a widely used medicinal plant, *Bupleurum* has exhibited a variety of biological activities. With the development of modern pharmacology, *Bupleurum* exerts significant biological effects across various domains, including anti-tumor, anti-inflammatory, anti-depressant, anti-aging, anti-epileptic, anti-pyretic, anti-viral properties, etc. These discoveries not only deepen our understanding of the medicinal value of *Bupleurum* but also establish a scientific foundation for its potential clinical applications (Figure 2).

### 4.1. Anti-Tumor

**Figure 3:**
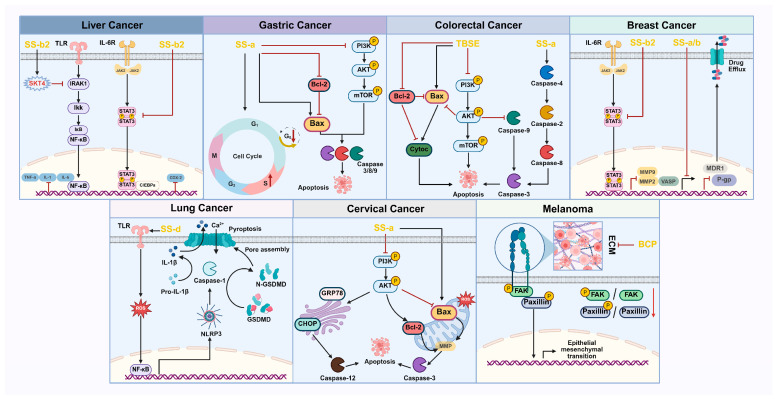
Anti-tumor Effects of *Bupleurum*. Integrative diagram summarizing the principal anti-cancer mechanisms of *Bupleurum* constituents in hepatocellular carcinoma (HCC), gastric cancer (GC), colorectal cancer (CRC), breast cancer, non-small-cell lung cancer (NSCLC), cervical cancer and melanoma. For each cancer type, the key active components (e.g., saikosaponin a/b2/d, total saponins, and acidic polysaccharides) and modulated targets/pathways are indicated (e.g., STK4/IRAK1→NF-κB, COX-2, PI3K/AKT/mTOR, STAT3, ROS-mediated apoptosis, NLRP3–caspase-1–GSDMD pyroptosis, and β1-integrin–FAK/paxillin adhesion signaling). Up- and down-regulations are denoted by ↑/↓. Abbreviations: STK4—serine/threonine kinase 4; IRAK1—interleukin-1 receptor-associated kinase 1; COX-2—cyclooxygenase-2; ROS—reactive oxygen species; NLRP3—NLR family pyrin domain containing 3; GSDMD—gasdermin-D; FAK—focal adhesion kinase.

Cancer is the leading cause of death worldwide [27]. When the tissue of a tumor invades nearby healthy tissue and leads to the formation of secondary tumors (metastases) in different organs, it can result in cancer. The incidence and mortality rates of malignant tumors are increasing, posing a significant threat to human health and becoming a public health issue of global concern. To date, most anti-tumor treatments have utilized small molecule drugs [28], which have achieved considerable success in saving the lives of cancer patients. However, the clinical application of anti-tumor drugs frequently encounters the issue of drug resistance [29]. Therefore, it is crucial to investigate and develop alternative drugs that are highly effective and have minimal side effects in order to inhibit cancer progression [30]. An integrated schematic of these anti-tumor mechanisms is provided in Figure 3.

*Bupleurum* contains a variety of active components, including triterpenoid saponins such as saikosaponin A [31,32,33,34], saikosaponin B [35], saikosaponin B2 [36,37], and saikosaponin D [38,39], as well as acidic polysaccharides [40,41] and the extract of total saikosaponins [42]. These components can target and regulate signaling pathways such as NF-κB, STK4/IRAK1, PI3K/AKT/mTOR, and STAT3, thereby inducing apoptosis and cell cycle arrest in cancer cells. *Bupleurum* extracts and their active components have demonstrated promising prospects for application in the treatment of various malignant tumors, including liver cancer, gastric cancer, colon cancer, breast cancer, lung cancer, cervical cancer, and melanoma. These findings not only confirm the significant antitumor effects of *Bupleurum* but also provide important theoretical foundations and clinical bases for the development of novel natural antitumor drugs.

#### 4.1.1. Anti-Liver Cancer

Liver cancer has a high incidence rate and is closely associated with chronic infections of the hepatitis B virus (HBV) or hepatitis C virus (HCV) [43,44]. These viral infections result in chronic viral hepatitis, which leads to persistent inflammation and damage to the liver, ultimately causing liver fibrosis and cirrhosis, and eventually resulting in liver cancer.

Chanhao Lei et al. [36] experimentally investigated the effects of Saikosaponin B2 (SS-b2) on primary liver cancer (PLC), the development of which is closely associated with chronic liver inflammation and the loss of related tumor suppressor genes. The research results indicate that in in vivo experiments, with an increase in the dose of SS-b2, the reduction in the expression of alanine aminotransferase (ALT) in mouse serum increased from 30.00% to 36.11%, the reduction in the expression of aspartate aminotransferase (AST) increased from 16.67% to 33.33%, and the increase in the level of lactate dehydrogenase (LDH) increased from 7.18% to 21.88%. Meanwhile, the positive chemotherapeutic agent doxorubicin (DOX) can reduce the expression of ALT by 50.00%, reduce the expression of AST by 38.89%, and lower the level of LDH by 25.00%. This indicates that SS-b2 has a substantial protective effect against liver damage. Furthermore, SS-b2 effectively enhances liver function in PLC mice. As the dosage increased, the number of liver surface nodules significantly decreased, illustrating a dose-dependent therapeutic effect. In histology, typical cancer nest structures and a significant infiltration of inflammatory cells were observed in the liver tissues of mice in the model group. Following treatment with SS-b2 and DOX, there was a notable reduction in the proliferation of cancer cells, the number of cancer nests, and the overall severity of cancer. Additionally, the degree of inflammatory cell infiltration and serum alpha-fetoprotein (AFP) levels also decreased. Concurrently, the infiltration of inflammatory cells decreased, and with increasing doses of SS-b2, the reduction in the expression level of serum alpha-fetoprotein (AFP) increased from 15.15% to 57.58%. The experiment employed immunohistochemical methods to detect the Ki67 antibody. The results demonstrated that after administration of SS-b2 and DOX, both the area of Ki67-positive staining and the number of Ki67-positive cells were reduced. Notably, SS-b2 increased the reduction in the number of Ki67-positive cells from 70.00% to 90.00%, confirming that SS-b2 effectively inhibits the malignant proliferation of liver cancer cells.

The incidence of liver cancer is closely associated with the expression of STK4 in immune cells [45]. Macrophages, which are the most abundant immune cells within the tumor microenvironment, participate in tumor development and can promote inflammatory responses, fibrosis, and cancer progression [46,47]. IRAK1 and NF-κB play an important role in regulating the production of proinflammatory cytokines in liver tumor cells and promoting cell proliferation. Both in vivo and in vitro experiments have demonstrated that SS-b2 can inhibit the expression levels of IL-1β, IL-6, and TNF-αmRNA. In in vivo experiments, as the dose of SS-b2 increased, the increase in STK4 protein expression in the liver tissue of LC mice rose from 44.44% to 55.56%, while the reduction in IRAK1 protein expression increased from 33.33% to 41.67% and the reduction in phosphorylated NF-κB p65 protein expression increased from 7.69% to 53.85%. In in vitro experiments, SS-b2 has also exhibited dose dependency; the increase in STK4 protein expression in HepG2 liver cancer cells rose from 23.08% to 84.62%, while the reduction in IRAK1 protein expression increased from 22.73% to 40.91% and the reduction in phosphorylated NF-κB p65 protein expression increased from 12.00% to 52.00%. In conclusion, SS-b2 impedes PLC by enhancing STK4 expression, downregulating IRAK1/NF-κB signaling, and decreasing the release of proinflammatory cytokines. Thus, SS-b2 has the potential to serve as a novel therapeutic agent targeting the STK4/IRAK1 pathway, thereby opening new avenues for the treatment of PLC.

*Bupleurum* saponin D (SS-d), an active compound extracted from *Bupleurum*, exhibits significant anti-cancer activity. Mudan Ren et al. [38] investigated the inhibitory effects of SS-d on two human liver cancer cell lines, SMMC-7721 and HepG2. Various in vitro techniques were employed, including the MTT assay, annexin-V-FITC/PI detection, Western blotting, immunohistochemistry, and qRT-PCR, to elucidate its mechanisms of action as a therapeutic agent for liver cancer. The research results showed that in IL-6-stimulated SMMC-7721 cells, with increasing doses of SS-d the reduction in p-STAT3 expression increased from 44.00% to 80.00%, the reduction in C/EBPβ expression increased from 39.13% to 73.91%, and the reduction in COX-2 expression increased from 24.14% to 79.31%. Similarly, in HepG2 cells, the reduction in p-STAT3 expression increased from 36.00% to 84.00%, the reduction in C/EBPβ expression increased from 37.93% to 79.31%, and the reduction in COX-2 expression increased from 36.67% to 80.00%. This demonstrates that SS-d exerts its anti-hepatoma effects by dose-dependently inhibiting the p-STAT3/C/EBPβ signaling pathway and significantly reducing the expression of cyclooxygenase (COX)-2. Meanwhile, SS-d exhibited a significant dose-dependent inhibitory effect on cell proliferation, achieving an inhibition rate of 35.00%, and significantly increased the occurrence of cell apoptosis. During this process, the expression of the pro-apoptotic protein Bax increased, while the expression of the anti-apoptotic protein Bcl-2 decreased. Furthermore, the study demonstrated that SS-d can inhibit the high expression of cell division protein kinase 6 (CDK6) and cyclin B1, thereby further suppressing the proliferation of liver cancer cells. In conclusion, SS-d effectively controls the proliferation of hepatocellular carcinoma cells by inhibiting the p-STAT3/C/EBPβ signaling pathway and reducing the expression of COX-2, thereby reinforcing its potential application in the treatment of hepatocellular carcinoma.

In the past few decades, the medicinal value of natural polysaccharides has received widespread attention from academics and related researchers. Shuyuan Shi et al. [40] extracted a water-soluble acidic polysaccharide (BCP) from *Bupleurum*, which primarily consists of rhamnosaccharide, arabinose, galactose, glucose, and galacturonic acid. The H22 tumor-bearing mouse model was employed to investigate the anti-tumor activity of BCP. The experimental results revealed that H22 tumor-bearing mice exhibited notable symptoms, including loss of appetite, reduced movement, and poor mental state. However, following BCP administration, these symptoms improved, suggesting that BCP has the potential to inhibit the growth of hepatocellular carcinoma cells. However, following the administration of BCP, the relevant symptoms improved, indicating that BCP has the potential to inhibit the growth of hepatocellular carcinoma cells. Additionally, the results of propidium iodide (PI) staining experiments demonstrated that BCP at concentrations of 100 mg/kg and 300 mg/kg could effectively induce apoptosis in H22 solid tumor cells. Compared to the model group, the number of cells in the G0/G1 and G2/M phases decreased (*p* < 0.05), while the number of cells in the S phase increased from 15.97% to 18.98% and 38.69%, respectively. This further demonstrates that BCP can induce apoptosis by blocking H22 solid tumor cells during the S phase of the cell cycle. Therefore, BCP shows promise as a safe and effective natural anti-hepatitis cancer drug in the medical field.

In summary, the treatment of liver cancer requires reasonable planning based on the different stages of the disease. For patients with early-stage liver cancer, surgical resection is the preferred treatment method; for patients with advanced stages, chemotherapy is the optimal treatment choice. The above research not only expands the potential application of *Bupleurum* in anti-liver cancer but also lays the foundation for the development of new anti-liver cancer therapeutic drugs, providing new possibilities for optimizing liver cancer treatment strategies.

#### 4.1.2. Anti-Gastric Cancer

Gastric cancer (GC) is one of the leading causes of cancer-related deaths worldwide [48]. Although significant progress has been made in the treatment of gastric cancer, including surgery, chemotherapy, targeted therapy and immune checkpoint inhibitors, the prognosis for gastric cancer is still poor, causing a large socio-economic burden [49,50].

To investigate the inhibitory effects of *Bupleurum* saponin A (SS-a) on gastric cancer cell lines including HGC-27, AGS, and MKN-28, Chao Wang et al. [31] employed both the MTT assay and cloning method for detection. The detection revealed that after the administration of SS-a, the viability of gastric cancer cells significantly decreased, and their proliferation rate was reduced by 90%. Furthermore, the study revealed that after the administration of SS-a, the proportion of HGC-27 cells in the S phase increased from 20.00% to 60.00%, while the proportion in the G0/G1 phase decreased from 70.00% to 30.00%. For AGS cells, the proportion in the S phase increased from 45.00% to 65%, and the proportion in the G0/G1 phase decreased from 50.00% to 30.00%. In MKN-28 cells, the proportion in the S phase increased from 29.00% to 70.00%, and the proportion in the G0/G1 phase decreased from 70.00% to 25.00%. These results imply that SS-a promotes apoptosis by inhibiting progression through the S phase of the cell cycle. In addition, the experiment also deeply explored the mechanism of SS-a action on gastric cancer cells. The study results showed that after SS-a administration, the expression level of the pro-apoptotic protein Bax in MKN-28 cells significantly increased, while the expression level of the anti-apoptotic protein Bcl-2 significantly decreased (*p* < 0.05). In addition, SS-a reduced the p-PI3K/PI3K ratio by 47.37%, the p-AKT/AKT ratio by 80.77%, and the p-mTOR/mTOR ratio by 63.20%. In summary, SS-a promotes the apoptosis of gastric cancer (GC) cells and suppresses their proliferation by inhibiting the PI3K-AKT signaling pathway and regulating the levels of Bax and Bcl-2 proteins. This further demonstrates that SS-a holds significant potential as a natural therapeutic agent in the treatment of gastric cancer.

Tae Woo Kim et al. [32] investigated the toxic effects of *Bupleurum* saponin A (SS-a) on human gastric cancer (GC) cells by detecting cell activity. Experimental results indicate that SS-a can inhibit the vitality of six human gastric cancer cell lines, including AGS, SNU-638, SNU-216, MKN-74, MKN-7, and NCI-N87. To further investigate the inhibitory effects of SS-a on gastric cancer cells, AGS cells were utilized to establish a mouse model of gastric cancer xenografts. The results demonstrated that, compared to the control group, tumor volume in the mice was significantly reduced after SS-a administration. Western blot analyses found that the expression levels of proapoptotic proteins PARP, caspase-3, caspase-8 and caspase-9 in AGS and MKN-74 cells after SS-a administration were enhanced, which shows that SS-a can induce GC cell apoptosis. Moreover, the existing literature suggests that endoplasmic reticulum (ER) stress can trigger the death of cancer cells and help overcome resistance to radiation therapy [51]. Further experiments through Western blot analysis revealed that after SS-a administration, the band intensity of glucose-regulated protein 78 (GRP78) in AGS cells increased from 0.2 to 1 over time, and the band intensities of phosphorylated protein kinase R-like endoplasmic reticulum kinase (p-PERK), phosphorylated eukaryotic initiation factor 2α (p-eIF2α), and activating transcription factor 4 (ATF4) all increased from 0 to 1. In MKN-74 cells, the band intensities of GRP78, p-PERK, eIF2, and ATF4 proteins also showed a similar trend. Meanwhile, SS-a enhanced the release of lactate dehydrogenase (LDH), the activity of caspase-3, and the release of intracellular Ca^2+^. This suggests that SS-a induces apoptosis of GC cells by activating the ER stress pathway. In addition, colony formation assays, cell viability tests, and Western blot analyses were conducted. The results showed that the cell viability of radiation-resistant GC cells (AGSR and MKN-74R) decreased after SSA administration (*p* < 0.05). The results further show that SS-a has anti-cancer effects on radiation-resistant GC cells. Therefore, SS-a shows potential therapeutic efficacy against GC cells and, when combined with radiotherapy, could serve as a promising strategy for gastric cancer treatment.

In summary, *Bupleurum* saponin a has an inhibitory effect on gastric cancer cells, thereby providing both a theoretical foundation and practical value for the future application of *Bupleurum* in the treatment of gastric cancer.

#### 4.1.3. Anti-Colon Cancer

Colon cancer, as one of the most common malignant tumors in the digestive tract, is primarily treated with surgical intervention [52]. However, the clinical field still faces pressing challenges such as numerous postoperative complications, limited treatment options for elderly patients, significant side effects of chemotherapy, and the tendency for drug resistance. Therefore, the discovery of new anti-colon cancer drugs is both necessary and urgent.

To investigate the inhibitory effect of *Bupleurum* total saponin extract (TBSE) on the proliferation of colon cancer cells, Xiaojing Zhang et al. [42] conducted in vitro experiments that revealed a significant reduction in the survival rates of SW480 and SW620 cells following 24 h of TBSE treatment (*p* < 0.01). The treated cells exhibited abnormal morphology, including cytoplasmic coagulation and partial adhesion loss. Flow cytometry analysis revealed that the apoptosis rates of SW480 cells and SW620 cells after TBSE administration were 48.47% and 36.13%, respectively. Western Blot and RT-PCR results showed that after TBSE administration, the ratio of pro-apoptotic protein Bax/β-actin in SW480 cells increased by 41.54%, the ratio of caspase-3/β-actin increased by 125.00%, the ratio of caspase-9/β-actin increased by 110.00%, and the ratio of anti-apoptotic protein Bcl2/β-actin decreased by 110.00%. In SW620 cells, the ratio of Bax/β-actin increased by 216.67%, the ratio of caspase-3/β-actin increased by 133.33%, the ratio of caspase-9/β-actin increased by 29.31%, and the ratio of Bcl2/β-actin decreased by 50.00%. These findings suggest that TBSE induces apoptosis in human colon cancer SW480 and SW620 cells by regulating the balance of Bax/Bcl2 and caspase-9/caspase-3. Additionally, the study found that after TBSE administration, the expression levels of PI3K in SW480 cells decreased by 53.00%, Akt by 54.00%, and mTOR by 59.18%. In SW620 cells, the expression levels of PI3K decreased by 80.00%, Akt by 50.00%, and mTOR by 51.00%. In summary, TBSE effectively inhibits colon cancer cell proliferation and induces apoptosis by suppressing the activation of the PI3K/Akt/mTOR pathway. Therefore, TBSE is anticipated to be a potential therapeutic agent for colon cancer.

Su Jin Kang et al. [33] used a colon cancer xenograft model and in vitro experiments with human colon cancer cells to investigate the inhibitory effects of *Bupleurum* saponin A (SS-a) on colon cancer. The study demonstrated a significant reduction in tumor volume in mice following SS-a administration, indicating its efficacy in inhibiting tumor growth. Furthermore, SS-a was found to activate the expression of apoptosis-inducing proteins, including caspase-4, caspase-2, caspase-8, and caspase-3, in colon cancer cells. The investigation also explored the effect of caspase-4 activation and the activation sequences of caspase-2, caspase-8, and caspase-3 on the apoptosis of human colon cancer LoVo and SW480 cells. The results showed that after SS-a administration, the activities of caspase-2, caspase-8, and caspase-3 in LoVo cells and SW480 cells were enhanced, with the proportion of cells in the sub-G1 phase being 61.7% and 56.2%, and the proportion of cells with nuclear condensation/fragmentation being 53.7% and 48.6%. However, after the addition of the caspase-4 inhibitor, their activities were weakened, with the proportion of cells in the sub-G1 phase being 19.2% and 18.8%, and the proportion of cells with nuclear condensation/fragmentation being 14.2% and 12.8%. The results of the western blot analysis further demonstrated that after inhibiting caspase-4, SS-a reduced the expression levels of caspase-3 by 62.50%, caspase-8 by 54.00%, and caspase-2 by 54.72%. In summary, SS-a induces apoptosis by facilitating the sequential activation of caspase-4, caspase-2, caspase-8, and caspase-3, thereby inhibiting the proliferation of colon cancer cells. Consequently, SS-a demonstrates significant potential in the adjuvant treatment of colon cancer.

To sum up, *Bupleurum* has demonstrated great significance in the treatment of colon cancer, providing new ideas and possibilities for treating this disease. Furthermore, it lays a solid foundation for future research and clinical application in colon cancer treatment, further emphasizing the potential of *Bupleurum* in cancer treatment.

#### 4.1.4. Anti-Breast Cancer

Breast cancer is the most common malignant tumor in women [53] and the leading cause of cancer-related deaths among women globally, with cancer metastasis being a critical factor in patient mortality. Therefore, the development of novel and more effective treatments for breast cancer is crucial and urgent.

To investigate the impact of *Bupleurum* saponin B2 (SS-b2) on MCF-7 breast cancer cells, Qing Ma et al. [37] conducted MTT cell proliferation experiments, RT-qPCR, and Western blotting to systematically analyze its underlying mechanism of action. The experimental results showed that after the administration of SS-b2, the proliferation of MCF-7 breast cancer cells decreased by 51.00%. At the same time, the expression of STAT3 and vasodilators stimulating phosphorylated protein (VASP) mRNA was found to be upregulated in MCF-7 breast cancer cells. Further studies revealed that after SS-b2 administration, the expression of c-myc and cyclin D1, which are associated with cell proliferation in the STAT3 signaling pathway in MCF-7 cells, was downregulated (*p* < 0.05). Simultaneously, with increasing doses, SS-b2 induced a reduction in pSTAT3 protein expression from 21.88% to 79.17%, a decrease in STAT3 protein expression from 10.00% to 30.00%, and a decline in VASP protein expression from 2.50% to 43.75%. The expression of matrix metallopeptidase (MMP) 2 was reduced by 60.00%, and the expression of MMP9 was decreased by 25.00%. These findings indicate that SS-b2 effectively suppresses the proliferation of MCF-7 breast cancer cells by inhibiting the activation of STAT3 and reducing the expression levels of VASP, MMP2, and MMP9. Therefore, SS-b2 can be used as an adjuvant drug which shows good application prospects in molecularly targeted treatment of breast cancer.

Dan Wang et al. [35] investigated the effects of *Bupleurum* saponin A (SS-a) and *Bupleurum* saponin B (SS-b) on doxorubicin-resistant breast cancer cells (MCF-7ADR) in doxorubicin (Dox) sensitivity through in vitro experiments. The results showed that after administration of 2.5 μg/mL SS-a and 5.0 μg/mL SS-b, the half-maximal inhibitory concentration (IC50) of Dox significantly decreased from 210.96 μg/mL to 84.93 μg/mL and 59.72 μg/mL, respectively, indicating that SS-a and SS-b can enhance the sensitivity of MCF-7ADR cells to Dox. Furthermore, the study explored the role of SS-a and SS-b in a multidrug resistance (MDR) reversal mechanism involving P-glycoprotein (P-gp). The results showed that with the increase in SS-a concentration, the reduction in P-gp expression in MCF-7ADR cells increased from 27.5% to 39.8%, and the reduction in MDR1 expression increased from 19.00% to 35.00%. SS-b increased the reduction in P-gp expression in MCF-7ADR cells from 21.48% to 29.50%, and the reduction in MDR1 expression from 8.00% to 31.00%, thereby reversing multidrug resistance (MDR). Consequently, SS-a and SS-b may serve as novel agents for reversing multidrug resistance, showing significant potential in breast cancer chemotherapy and holding important clinical implications.

The current treatment for breast cancer primarily involves surgical resection, supplemented by medical therapies such as radiotherapy, chemotherapy, endocrine therapy, and targeted therapy [54]. Although multidisciplinary comprehensive treatment has improved survival rates, treatment-related adverse reactions remain prominent. The above research found that Saikosaponin A and Saikosaponin B2 can effectively inhibit breast cancer cells, which not only highlights the potential application of *Bupleurum* in breast cancer treatment but also provides important clinical value for the development of new anti-breast cancer drugs.

#### 4.1.5. Anti-Lung Cancer

Lung cancer is one of the most prevalent cancers worldwide and remains the leading cause of cancer-related mortality [55]. Among the various types of lung cancer, non-small cell lung cancer (NSCLC) accounts for 80% to 85% [56] of cases.

To investigate whether *Bupleurum* saponin D (SS-d) can induce pyroptosis in NSCLC, Mengqing Chen et al. [39] administered varying concentrations of SS-d to A549 and HCC827 cell lines through in vitro experiments. The experimental results indicate that following the administration of 10 μM and 20 μM SS-d, there was a significant increase in the number of balloon-like cells in both A549 and HCC827 cells (*p* < 0.01). Meanwhile, the content of lactate dehydrogenase (LDH) in the cell supernatant increased by 237.50% and 178.57%. These findings suggest that SS-d effectively induces pyroptosis in lung cancer cells. The experiment further employed immunofluorescence and Western blotting techniques to investigate the pathways through which SS-d induces pyroptosis in lung cancer cells. The study results showed that with the increase in dosage, SS-d could elevate the NLRP3/GAPDH ratio in HCC827 cells from 90.00% to 250.00%, the GSDMD-N/GAPDH ratio from 75.00% to 140.00%, and the cleaved-caspase-1/caspase-1 ratio from 10.00% to 80.00%. In A549 cells, the increase in the NLRP3/GAPDH ratio rose from 198.00% to 210.00%, the increase in the GSDMD-N/GAPDH ratio escalated from 139.00% to 250.00%, and the increase in the cleaved-caspase-1/caspase-1 ratio climbed from 50.00% to 160.00%, indicating that SS-d could enhance the expression of pyroptosis-related proteins GSDMD, NLRP3, and cleaved caspase-1 in HCC827 cells (*p* < 0.05), thereby activating the NLRP3/caspase-1/GSDMD pathway. Additionally, flow cytometry results revealed that following SS-d administration, a large amount of reactive oxygen species (ROS) was generated in both HCC827 and A549 cells, with a significant increase in DCFH-DA fluorescence intensity (*p* < 0.01). The study also found that SS-d administration led to an increase in the expression levels of NF-κB acetylated proteins in lung cancer cells by 180.00% and 136.84%, respectively (*p* < 0.05).

In summary, SS-d induces apoptosis in lung cancer cells by promoting the accumulation of ROS and activates the NF-κB/NLRP3/caspase-1/GSDMD pathway, thereby exhibiting an anti-non-small cell lung cancer effect. This finding provides valuable evidence and support for the development of new therapeutic drugs.

#### 4.1.6. Anti-Cervical Cancer

Cervical cancer is the most prevalent malignant tumor responsible for illness and fatalities among women in clinical practice, ranking seventh overall among all types of malignant tumors [57]. Jikun Du et al. [34] investigated the effect of SS-a on cervical cancer cells (HeLa) through in vitro experiments. The results from flow cytometry indicated that the apoptosis rates of HeLa cells following SS-a administration at concentrations of 5 μM, 10 μM, and 15 μM were 6.96%, 18.32%, and 48.80%, respectively. These findings demonstrate that SS-a effectively induces apoptosis in HeLa cells. Further investigations have revealed that SS-a induces a decrease in mitochondrial membrane potential (MMP) within HeLa cells, and a significant increase in reactive oxygen species (ROS) levels (*p* < 0.05). Western blot analysis demonstrated that SS-a upregulates the expression of pro-apoptotic proteins, including Bax and caspase-3, in HeLa cells, while simultaneously downregulating the anti-apoptotic protein Bcl-2. Additionally, it increases the expression of the endoplasmic reticulum stress-related protein GRP78 by 60.00%, CHOP by 75.00%, and caspase-12 by 50%.

In summary, SS-a induces apoptosis in HeLa cells by activating both the mitochondrial pathway and the endoplasmic reticulum stress pathway, thereby exerting an anti-cervical cancer effect. Consequently, SS-a is anticipated to serve as an adjunctive drug in the treatment of cervical cancer.

#### 4.1.7. Anti-Melanoma

Melanoma is a common form of cancer that can occur in multiple sites, including the skin, iris, and rectum [58]. It is characterized by its aggressive nature and high metastatic potential, allowing it to spread rapidly throughout the body. For patients with metastatic malignant melanoma, the prognosis is grave, with a 5-year survival rate of less than 15% [59]. Therefore, interfering with tumor metastasis has been recognized as a crucial strategy for improving the treatment of melanoma.

Extracellular matrix (ECM) adhesion plays a crucial role in tumor metastasis, with fibronectin serving as the most prevalent integrin ligand within the ECM [60]. Haibin Tong et al. [41] extracted *Bupleurum* polysaccharide (BCP) from *Bupleurum* and identified its composition through gas chromatography, revealing that it primarily consists of four monosaccharides: arabinose, xylose, mannose, and glucose. Concurrently, in vitro experiments demonstrated that BCP effectively inhibits the ECM-mediated adhesion of A375 cells. In A375 cells, β1 integrin serves as the primary fibronectin receptor [61], while the GST-FNIII9-11 protein contains the RGD motif that binds to β1 integrin. The experiment revealed that immunoprecipitated β1 integrin exhibits high affinity with GST-FNIII9-11. However, after administration of 100 and 400 μg/mL BCP, the blocking rates of the affinity between the two were 75.2% and 93.8%, respectively, indicating that BCP can block the interaction between β1 integrin and its physiological ligand fibronectin. Additionally, the impact of BCP on signaling pathways associated with β1 integrin in A375 cells was further investigated using Western blot analysis. The results showed that with the increase in dosage, the reduction in the ratio of p-FAK to total focal adhesion kinase (FAK) in A375 cells after BCP administration increased from 40.00% to 65.00%, and the reduction in the ratio of p-paxillin to total paxillin increased from 25.00% to 75.00%. In summary, BCP can down-regulate the downstream signal of β1 integrin, such as FAK and paxillin, thereby inhibiting the adhesion of A375 cells to fibronectin and exerting an anti-melanoma effect. Consequently, BCP may be utilized as a therapeutic agent to interfere with melanoma metastasis.

**Figure 4:**
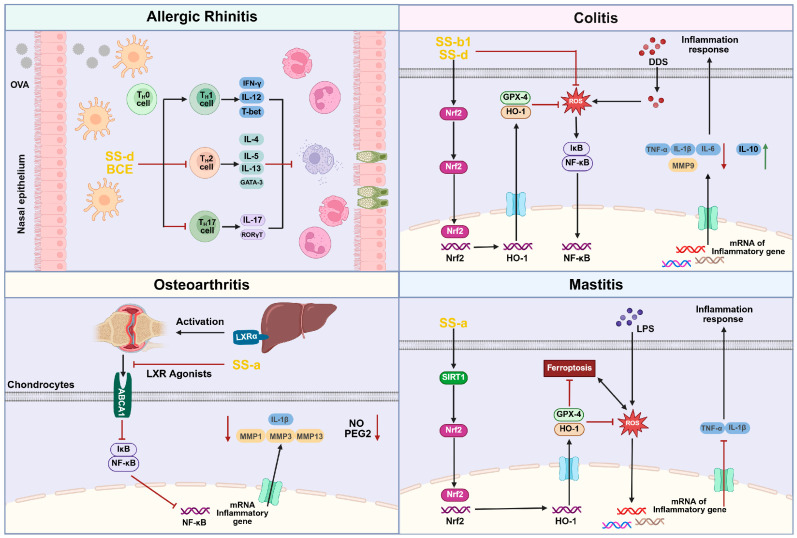
Anti-inflammatory effects of *Bupleurum*. Schematic summary of anti-inflammatory mechanisms reported for *Bupleurum* constituents. The diagram highlights the following: (i) inhibition of the NF-κB pathway (reduced nuclear translocation/acetylation of NF-κB and down-regulation of pro-inflammatory mediators); (ii) activation of the SIRT1/Nrf2 axis that augments antioxidant defense and mitigates oxidative stress; (iii) suppression of ferroptosis-related damage; and (iv) anti-allergic modulation (e.g., reduction of OVA-specific antibodies). Up- and down-regulations are indicated by ↑/↓ and inhibitory “⊥” symbols. Abbreviations: NF-κB—nuclear factor-κB; SIRT1—sirtuin-1; Nrf2—nuclear factor erythroid 2–related factor 2; ROS—reactive oxygen species; OVA—ovalbumin; Ig—immunoglobulin.

*Bupleurum* has demonstrated significant anti-cancer activity across various malignancies. The evidence spans multiple cancer types such as hepatocellular carcinoma (HCC), gastric cancer, colorectal cancer, breast cancer, non-small-cell lung cancer (NSCLC), cervical cancer, and melanoma. Table 2 summarizes *Bupleurum*’s anti-tumor effects organized by cancer type, highlighting the experimental models used, the key mechanisms of action (e.g., induction of apoptosis, cell cycle arrest, and inhibition of pro-survival signaling pathways), and the main molecular targets modulated. This overview underscores the multi-target nature of *Bupleurum*’s components (like saikosaponins) in suppressing tumor growth and inducing cancer cell death. In summary, this study not only broadens the application scope of *Bupleurum* in the anti-tumor domain, but also provides a new perspective and entry point for research on tumor treatment. Additionally, the study presents a comprehensive discussion of the biological mechanisms of *Bupleurum*, which in turn generates innovative ideas for the development of anti-cancer treatment strategies (Figure 4).

**Table 2:** Anti-tumor effects of *Bupleurum*: key mechanisms and targets in various cancer models.

Effect and Disease	Adopted Model	Main Mechanisms	Main Targets	References
HCC	1. PLC mice treated with SS-b2 or doxorubicin2. SMMC-7721 and HepG2 cells exposed to SS-d3. H22 hepatoma-bearing mice given BCP	SS-b2 raises STK4 expression, which in turn lowers IRAK1 and phosphorylated NF-κB p65, diminishes IL-1β/IL-6/TNF-α production and restrains tumor growth. SS-d blocks the p-STAT3/C-EBPβ axis, decreases COX-2 and reverses the Bax/Bcl-2 ratio, thereby activating caspase-3/9-mediated apoptosis. BCP arrests the cell cycle in the S phase and triggers apoptosis in tumors.	STK4, IRAK1, NF-κB p65, STAT3, COX-2, Bax, Bcl-2, caspase-3/9	[36,38,40]
Gastric cancer	1. HGC-27, AGS, MKN-28 cells treated with SS-a 2. AGS xenografts (including radio-resistant AGS-R and MKN-74-R)	SS-a blocks the PI3K–Akt–mTOR cascade, elevates Bax and lowers Bcl-2, leading to S-phase arrest and apoptosis. It simultaneously provokes ER stress (GRP78–PERK–eIF2α–ATF4–CHOP), activates caspase-3/8/9 and enhances radiosensitivity in resistant GC cells.	PI3K, Akt, mTOR, Bax, Bcl-2, GRP78, PERK, CHOP, caspase-3/8/9	[31,51]
Colorectal cancer	1. SW480 and SW620 cells exposed to TBSE 2. LoVo and SW480 cells and xenograft mice treated with SS-a	TBSE suppresses PI3K–Akt–mTOR signaling, shifts the Bax/Bcl-2 balance and activates caspase-9/3, culminating in apoptosis. SS-a sequentially activates caspase-4, then caspase-2/8/3, thereby inducing apoptosis and reducing tumor volume in vivo.	PI3K, Akt, mTOR, Bax, Bcl-2, caspase-4/2/8/3/9	[33,42]
Breast cancer	1. MCF-7 cells (SS-b2) 2. Adriamycin-resistant MCF-7/ADR cells (SS-a, SS-b)	SS-b2 inhibits STAT3 phosphorylation, down-regulates VASP and MMP-2/9, and thus curbs proliferation and migration. SS-a and SS-b repress MDR1 mRNA and P-gp, lowering the IC_50_ of doxorubicin in resistant cells.	STAT3, VASP, MMP-2/9, MDR1, P-gp	[35,37]
NSCLC	A549 and HCC827 cells treated with SS-d	SS-d accumulates ROS, acetylates NF-κB, and activates the NLRP3–caspase-1–GSDMD axis, leading to pyroptotic cell death.	ROS, NF-κB, NLRP3, caspase-1, GSDMD	[39]
Cervical cancer	HeLa cells treated with SS-a	SS-a decreases mitochondrial membrane potential, elevates ROS, and up-regulates Bax, caspase-3, and ER stress proteins (GRP78, CHOP, caspase-12), while suppressing PI3K/Akt, thereby inducing apoptosis.	Bax, Bcl-2, caspase-3/12, GRP78, CHOP, PI3K/Akt	[34]
Melanoma	A375 cells exposed to BCP	BCP disrupts β1-integrin binding to fibronectin, reduces phosphorylation of FAK and paxillin, and inhibits ECM-mediated adhesion, thereby impeding metastasis.	β1-integrin, FAK, paxillin	[41]

### 4.2. Anti-Inflammatory

**Figure 5:**
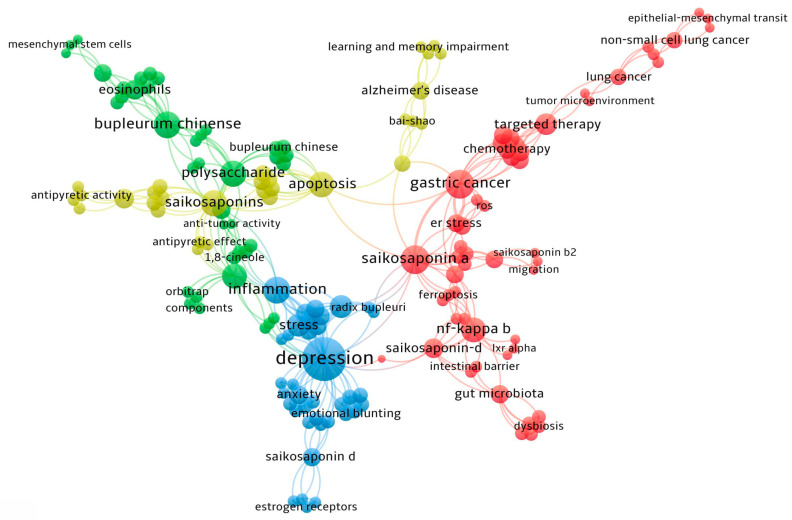
VOSviewer of *Bupleurum*. Network visualization generated with VOSviewer showing co-occurrence of author keywords in the *Bupleurum* literature. Node size reflects keyword frequency/importance; edges indicate co-occurrence strength; colors denote algorithm-derived clusters of related topics. This map helps identify core themes, emerging trends and cross-disciplinary links in *Bupleurum* studies.

Inflammation is a complex immune response and serves as a defense mechanism employed by the body to combat pathogens, toxic compounds, and various environmental stress factors [62]. However, if the autologous injury induced by inflammation is not addressed promptly, or if the irritant persists, it may lead to the development of associated pathological states. In such cases, immune cells will continue to be activated, resulting in further damage to the body’s health [63]. Although nonsteroidal anti-inflammatory drugs [64] and glucocorticoids are commonly used to treat inflammation, there are many adverse reactions and they should not be used long term. In this context, the development of novel anti-inflammatory drugs has become an urgent priority. The natural product *Bupleurum* has garnered significant attention due to its notable anti-inflammatory activity, and in-depth research into its anti-inflammatory mechanisms holds considerable value. Studies have shown that multiple *Bupleurum* components exert anti-inflammatory effects via distinct mechanisms; the principal pathways and targets are summarized in Figure 5. Among them, triterpenoid saponins exhibit significant anti-inflammatory properties: Saikosaponin D [65,66] demonstrates favorable therapeutic effects in animal models of allergic rhinitis and ulcerative colitis by regulating the NF-κB signaling pathway; Saikosaponin A [67,68] operates through a dual mechanism, inhibiting the NF-κB pathway on one hand and activating the SIRT1/Nrf2 signaling cascade on the other, thereby playing a role in the treatment of osteoarthritis and mastitis; Saikosaponin B1 [69] alleviates symptoms of colitis by blocking the ferroptosis pathway. Additionally, *Bupleurum* extract [70] can significantly improve the clinical symptoms of allergic rhinitis by modulating OVA-specific antibody levels.

#### 4.2.1. Anti-Rhinitis

Allergic rhinitis (AR) is an allergic inflammation of the nasal airway and is among the most prevalent chronic respiratory diseases worldwide [71,72]. It is characterized by sneezing, nasal congestion [73], itching and nasal leakage [74,75]. Antiallergic and antihistamine drugs can only alleviate the symptoms of the inflammatory response, and their effects are often short-lived. Therefore, it is crucial to explore and develop new treatment strategies for allergic rhinitis (AR).

Chun Hua Piao et al. [65] investigated the nasal friction and sneezing behavior of mice with allergic rhinitis (AR) induced by ovalbumin (OVA) to examine the inhibitory effects of *Bupleurum* saponin D (SS-d) on OVA-induced AR symptoms in mice. The results showed that after SS-d administration, the frequency of nasal rubbing and sneezing in mice decreased by 37.50% and 38.78%, respectively, indicating that SS-d can effectively alleviate AR-related symptoms. Further research revealed that SS-d treatment mitigates damage to the nasal mucosal epithelium in mice and inhibits the infiltration and hyperplasia of mast cells and goblet cells. Concurrently, tissue cell infiltration and tissue fibrosis in lung tissues were also improved. Furthermore, immunological test results showed that after SS-d treatment, the expression levels of OVA-specific IgE and IgG1 in mice decreased by 28.57% and 71.43%, respectively, while the expression level of IgG2a protein increased by 21.43%. This finding indicates that SS-d can inhibit the production of allergic mediators, thereby regulating the body’s immune response. In terms of the inflammatory mechanism, in nasal lavage fluid (NALF), SS-d reduced the expression of NF-κb by 75.44% and P-NF-κB by 8.00%. In lung homogenates, SS-d decreased the expression of NF-κb by 30.00% and P-NF-κB by 28.57%. This indicates that SS-d mitigates inflammation by inhibiting the NF-κb pathway, thereby further enhancing its anti-allergic effects. In summary, SS-d not only alleviates pathological changes in the nasal mucosa and lung tissue but also exerts therapeutic effects by modulating specific immune responses and inducing inflammatory signaling pathways. Therefore, SS-d is anticipated to serve as an adjunctive treatment for allergic rhinitis, providing novel insights and directions for the treatment of related diseases.

Thi Tho Bui et al. [70] investigated the anti-allergic and anti-inflammatory effects of *Bupleurum* extract (BCE) on allergic rhinitis (AR). In vivo experiments were performed using a mouse model of ovalbumin (OVA)-induced allergic rhinitis to evaluate the impact of BCE on the early allergic symptoms of AR. The results demonstrated that following BCE treatment, the frequency of friction and sneezing in mice was significantly reduced, which indicated that the symptoms of nasal allergies in mice were effectively relieved. Further studies revealed that BCE could reduce the infiltration of neutrophils and macrophages, as well as the number of eosinophils (*p* < 0.05), and decrease the number of inflammatory cells in the nasal lavage fluid of mice. With increasing doses of BCE, the reduction in the expression level of chemokine protein 2 (CCL24) in the serum of mice increased from 18.18% to 27.27%, which further improved the structure of the nasal mucosa. In terms of immune response, BCE can target and regulate the levels of OVA-specific antibodies, leading to an increase in the reduction of anti-OVA-specific protein IgE levels from 19.35% to 61.29%, an increase in the reduction of IgG1 levels from 21.05% to 26.32%, and an increase in the enhancement of IgG2a levels from 80.00% to 270.00%. Additionally, it was observed that following BCE administration, both mast cells and degranulated mast cells were reduced, as indicated by Giemsa staining (*p* < 0.05). Further analysis revealed that after oral administration of BCE, the expression levels of IL-4 in nasal lavage fluid decreased by 10.00%, IL-5 decreased by 12.00%, and IL-13 decreased by 58.12%, while the expression levels of IL-10 increased by 27.27%, IL-12 increased by 16.67%, and INF-γ increased by 25.00%. In summary, BCE can effectively alleviate the allergic symptoms associated with variant rhinitis. The mechanisms of its anti-allergic and anti-inflammatory effects include targeted regulation of OVA-specific antibody levels, reduction of mast cell activation, and modulation of the immune response of helper T cells. These research findings provide a theoretical basis for the clinical application of BCE in the treatment of AR.

In summary, *Bupleurum* has been shown to effectively inhibit early allergic symptoms in mice with allergic rhinitis, reduce the infiltration of inflammatory cells in the nasal cavity, and improve the structure of the nasal mucosa. These research results provide an important experimental foundation for the future development of new drugs for the treatment of allergic rhinitis.

#### 4.2.2. Anti-Colitis

Colitis is an inflammatory disease of the intestinal tract, with ulcerative colitis (UC) [76] being the most prevalent form. UC is classified as a nonspecific inflammatory bowel disease characterized by chronic and recurrent episodes that exclusively affect the colon and rectum [77]. Patients with UC commonly present with symptoms such as abdominal pain, weight loss, diarrhea, and bloody stools [78,79,80]. Potential causes of UC include abnormal immune responses, dysfunction of the intestinal barrier, environmental factors, individual susceptibility, and disorders of the intestinal microbiota.

This study investigates the therapeutic effects of *Bupleurum* saponin B1 (SS-b1) and *Bupleurum* saponin D (SS-d) on colitis. Huimei Hu et al. [69] established a zebrafish model of colitis induced by dextran sulfate sodium (DSS) for in vivo experiments. RT-qPCR analysis revealed that with increasing doses, SS-b1 could enhance the reduction in mRNA expression of matrix metalloproteinase 9 (MMP9) from 39.06% to 71.88%, interleukin-6 (IL-6) from 42.86% to 85.71%, interleukin-8 (IL-8) from 8.00% to 68.00%, and tumor necrosis factor-alpha (TNF-α) from 28.89% to 51.11%. SS-d could increase the reduction in MMP9 mRNA expression from 65.00% to 80.00%, decrease IL-6 mRNA expression by 87.10%, increase the reduction in IL-8 mRNA expression from 44.00% to 70.00%, and increase the reduction in TNF-α mRNA expression from 71.79% to 76.92%. Research indicates that inhibiting ferrodystrophy can effectively improve DSS-induced colitis. Three key indicators of ferrodystrophy include malondialdehyde (MDA), glutathione (GSH), and iron load [81]. Therefore, this study investigated the effects of SS-b1 and SS-d on ferrodynamics in detail. The results revealed that following the administration of SS-b1 and SS-d, the expression of prostaglandin-endoperoxide synthase 2 (PTGS2) in zebrafish tissues was downregulated, while the expression of ferritin light chain (FTH and FTL) was upregulated, and the expression level of MDA and iron load were reduced. Furthermore, the study found that with increasing doses, SS-b1 could elevate the increase in NRF2 mRNA expression from 25.00% to 50.00%, and the increase in HO-1 mRNA expression from 50.00% to 183.33%, while SS-d could raise the expression of NRF2 mRNA by 46.67%, and the increase in HO-1 mRNA expression from 100.00% to 233.33%. In summary, SS-b1 and SS-d inhibit ferrodystrophy by activating the NRF2/HO-1 pathway, demonstrating significant therapeutic effects on DSS-induced colitis which demonstrates the potential of SS-d in the treatment of colitis and its clinical application value.

Puze Li et al. [66] investigated the influence of *Bupleurum* saponin D (SS-d) on mice with ulcerative colitis (UC) induced by DSS. Following treatment with SS-d, the UC mice exhibited an increase in weight (*p* < 0.05), a decrease in the disease activity index (DAI) (*p* < 0.05), an increase in colon length (*p* < 0.05), and significant protection of colon tissue, with a notable reduction in inflammation. RT-qPCR detection revealed that in the colonic tissues of UC mice, SS-d could reduce the expression levels of pro-inflammatory factors TNF-α mRNA by 37.50%, IL-6 mRNA by 56.25%, and IL-1β mRNA by 79.41%, while increasing the expression level of anti-inflammatory factor IL-10 mRNA by 129.63%. In addition, SS-d can increase the expression level of IκB by 100.00% and decrease the expression level of p-IκB by 40.00%, indicating its inhibition of the activation of the NF-κB signaling pathway. Further studies revealed that SS-d (8 mg/kg/d) significantly increased the expression levels of colonic tissue mucin Muc1 mRNA by 128.57% and Muc2 mRNA by 55.88% and restored the protein levels of zonula occludens-1 (ZO-1) and Claudin-1 (*p* < 0.05), thereby protecting the intestinal barrier. In conclusion, SS-d alleviates DSS-induced ulcerative colitis (UC) through the suppression of NF-κB activation and the modulation of the intestinal microbiome. This establishes a solid medical and clinical foundation for SS-d as a drug candidate for the treatment of UC and suggests that it will play an important role in the clinical treatment of UC.

To summarize, *Bupleurum*, a traditional herb, exhibits a therapeutic effect on colitis. Future research should focus on exploring the specific mechanisms of *Bupleurum*, determining its optimal dosage, and investigating its combined application with other therapies to provide a more comprehensive therapeutic strategy for patients with colitis.

#### 4.2.3. Anti-Osteoarthritis

Osteoarthritis (OA) is a common form of arthritis characterized by the degradation and destruction of the cartilage matrix, alongside an inflammatory response from the chondrocytes [82,83]. Currently, the treatment of osteoarthritis primarily involves conservative medication and surgical interventions; however, these approaches may result in side effects and complications, such as gastrointestinal issues, allergic reactions, and nerve or vascular damage. Consequently, it is crucial to explore novel strategies for the treatment of OA.

To investigate the anti-inflammatory effect of *Bupleurum* saponin A (SS-a) on IL-1β-stimulated human osteoarthritis chondrocytes, Han Gao et al. [67] conducted in vitro experiments utilizing ELISA, Griess assays, and qRT-PCR detection techniques. Research has found that SS-a can inhibit the release of inflammatory mediators NO and PGE2 in human osteoarthritis chondrocytes stimulated by IL-1β and can increase the reduction in MMP1 expression from 22.00% to 80.00%, MMP3 expression from 35.00% to 80.00%, and MMP13 expression from 30.77% to 84.62%, thereby exerting anti-inflammatory effects. Furthermore, additional studies have revealed that SS-a can increase the reduction in the p-IkB/β-actin ratio from 30.00% to 81.00% and enhance the increase in LXRα expression from 137.50% to 525.00% in human osteoarthritis chondrocytes stimulated by IL-1β. In summary, SS-a treats IL-1β-induced chondrocyte inflammation by activating LXRα, inhibiting NF-κB activation, and reducing the release of IL-1β-induced inflammatory mediators.

In summary, *Bupleurum* and its extracts demonstrate potential application value in the treatment of osteoarthritis, providing more choices and more effective treatment methods for the treatment of osteoarthritis.

#### 4.2.4. Anti-Mastitis

Mastitis is a breast disease caused by infection with Staphylococcus aureus [84]. Lihua Zhao et al. [68] investigated the therapeutic effect of SS-a on mouse mastitis induced by Staphylococcus aureus through in vivo experiments to explore its clinical application value. The study found that SS-a can reduce the activity of myeloperoxidase (MPO) by 60.00%, decrease the expression level of TNF-α by 77.63%, and lower the expression level of IL-1β by 62.00%, indicating that SS-a significantly inhibits inflammatory responses and demonstrates anti-inflammatory properties. Meanwhile, the study found that SS-a could increase the occludin/β-actin ratio by 380.00%, the ZO-1/β-actin ratio by 650.00%, and the claudin-3/β-actin ratio by 425.00%, thereby ameliorating the damage to the blood-milk barrier and further enhancing its protective effect. In terms of antioxidant properties, SS-a demonstrates promising results. It effectively reduces Fe^2+^ levels in breast tissue and upregulates the expression of antioxidant factors, such as glutathione and glutathione peroxidase 4 (GPX4), while also decreasing the expression of PTGS2 and malondialdehyde (MDA), thereby improving ferroptosis. Furthermore, with the increase in SS-a dosage, the increase in the Nrf2/Lamin B ratio rose from 60.00% to 260.00%, the increase in the HO-1/β-action ratio rose from 135.29% to 429.41%, and the increase in the SIRT1/β-action ratio rose from 110.00% to 275.00%. This indicates that SS-a effectively inhibits infection by promoting the expression of SIRT1, Nrf2, and HO-1 in mammary tissue, thereby activating the SIRT1/Nrf2 signaling pathway. In summary, SS-a shows potential as an auxiliary drug for preventing and treating mastitis caused by Staphylococcus aureus.

*Bupleurum* exhibits broad anti-inflammatory properties in diverse disease models. Table 3 provides a consolidated view of its anti-inflammatory effects across conditions such as allergic rhinitis, ulcerative colitis, osteoarthritis, and mastitis. The table is organized by specific inflammatory condition, detailing how *Bupleurum* extracts or compounds reduce inflammation (for instance, by inhibiting NF-κB signaling, lowering pro-inflammatory cytokine levels, or preventing cell damage via ferroptosis) and listing the key molecular targets involved. This categorized summary highlights *Bupleurum*’s capacity to modulate immune responses and maintain barrier integrity in different tissues and organ systems. To sum up, *Bupleurum* has shown great potential as an anti-inflammatory and will also show important value in traditional and modern medical practice.

**Table 3:** Anti-Inflammatory Effects of *Bupleurum*: Key Mechanisms and Targets in Various Disease Models.

Condition	Adopted Model	Main Mechanisms	Main Targets	References
Allergic rhinitis	1. OVA-sensitized mice given SS-d 2. OVA-sensitized mice given BCE	SS-d and BCE both reduce sneezing and nasal rubbing, dampen eosinophil and mast-cell infiltration, lower IgE/IgG1 and Th2 cytokines, raise IgG2a and IL-10, and inhibit NF-κB signaling, improving nasal mucosal integrity.	NF-κB, IL-4/5/13, IgE, IgG1/2a, IL-10	[65,70]
Ulcerative colitis	1. DSS-induced zebrafish colitis (SS-b1, SS-d) 2. DSS-induced mouse colitis (SS-d)	SS-b1 and SS-d activate NRF2/HO-1, diminish lipid peroxidation and iron loading, and inhibit ferroptosis. SS-d also suppresses NF-κB, decreases TNF-α/IL-6/IL-1β, elevates IL-10, and recovers barrier proteins ZO-1, Claudin-1, Muc1/2.	NRF2, HO-1, TNF-α, IL-6/1β/10, ZO-1, Claudin-1, Muc1/2	[66,69]
Osteoarthritis	IL-1β-stimulated human chondrocytes treated with SS-a	SS-a up-regulates LXRα, blocks NF-κB activation, and reduces NO, PGE_2_, and MMP-1/3/13, alleviating chondrocyte inflammation.	LXRα, NF-κB, MMP-1/3/13, NO, PGE_2_	[67]
Mastitis	*S. aureus*-induced mouse mastitis treated with SS-a	SS-a activates SIRT1/Nrf2, raises HO-1, enhances tight-junction proteins, decreases Fe^2+^ and ferroptosis markers, and suppresses inflammatory cytokines, protecting the blood–milk barrier.	SIRT1, Nrf2, HO-1, ZO-1, Occludin, Claudin-3	[68]

### 4.3. Anti-Depression

As a common and severe mental illness, depression is characterized by persistent low mood, slowed thinking, loss of interest, reduced self-evaluation, and sleep problems, and has become a public health issue that threatens public mental health and imposes a significant socioeconomic burden [85,86]. The existing antidepressant medications, including tricyclic antidepressants, selective serotonin reuptake inhibitors (SSRIs), and monoamine oxidase inhibitors (MAOIs), although they have certain therapeutic effects, still present issues of inadequate treatment efficacy in some patients, and are accompanied by various side effects [87,88]. Given this, the development of new antidepressants with higher safety and better efficacy has become a key focus of current research.

Natural products have garnered widespread attention due to their potential antidepressant activity, providing new perspectives and resources for the development of antidepressant drugs [89]. Research has shown that multiple active components of *Bupleurum*, including *Bupleurum* extract [90], total saponin extract [91], as well as saikosaponin D [92,93] and A [94], all exhibit effects in improving depressive symptoms. These components exert their therapeutic effects through different mechanisms, inhibiting the inflammatory response associated with depression, activating the mTOR signaling pathway, promoting the ubiquitination process of the NLRP3 protein, and regulating the activity of the Tet1/DLL3/Notch signaling pathway.

Alterations in neurotransmitter levels and inflammatory markers are critical indicators of depression [95,96]. To investigate the effects of *Bupleurum* on neurotransmitter levels and inflammation, Baijin Chang et al. [90] administered *Bupleurum* to rats suffering from chronic unpredictable mild stress (CUMS). In vivo experimental results showed that after administration of *Bupleurum*, the level of serotonin (5-HT) in the rat brain increased by 133.33%, dopamine (DA) increased by 28.57%, and norepinephrine (NE) increased by 15.69%, while the level of cortisol (CORT) in serum decreased by 37.50%. Simultaneously, the levels of inflammatory marker interleukin-6 (IL-6) decreased by 50.00% and interleukin-1β (IL-1β) expression decreased by 48.00%. Further research has demonstrated that the protein kinase cAMP-activated catalytic subunit alpha (PRKACA) and cAMP-response element binding protein (CREB) play significant roles in inhibiting inflammatory responses, while cAMP is one of the most important metabolites. Compared to the model group, the cAMP levels showed a significant dose-dependent increase after administration of Chaihu. Meanwhile, with the increase in the dose of Chaihu extract, the increase in PRKACA expression rose from 39.66% to 48.66%, and the increase in CREB expression rose from 41.86% to 45.36%. Collectively, these findings suggest that *Bupleurum* can effectively upregulate the expression of PRKACA and CREB, enhance cAMP levels, mitigate the inflammatory response associated with depression, and consequently alleviate depressive symptoms.

Tiantian Gao et al. [92] administered SS-d treatment to mice subjected to chronic unpredictable mild stress (CUMS) and evaluated the depression-like behavior of these mice using the sucrose preference test (SPT), forced swimming test (FST), and tail suspension test (TST). The findings indicated that SS-d effectively improved the depressive behavior of the CUMS mice. Moreover, the research analyzed the hippocampus of the mice using Western blot testing and enzyme-linked immunosorbent assay (ELISA). The results indicated that 1 mg/kg of SS-d reduced the ratios of p-NF-κb/NF-κb and pMAPK/MAPK by 5.33% and 40.54%, respectively, and decreased the levels of the pro-inflammatory cytokines IL-1β by 20.00%, TNF-α by 30.00%, and IL-18 by 16.67%. These findings suggest that SS-d can inhibit the hippocampal inflammatory response in CUMS mice. To further investigate the impact of SS-d on NLRP3 inflammasomes in the hippocampus, Western blot analysis was employed to detect the expression of related proteins. The results indicate that with an increase in SS-d dosage, the increase in the NLRP3/GAPDH ratio rises from 22.22% to 48.89%, the increase in the apoptosis-associated speck-like protein containing a CARD (ASC)/GAPDH ratio rises from 28.00% to 60.60%, and the increase in the Cleaved-Caspase1/Caspase1 ratio rises from 36.00% to 70.00%, demonstrating that SS-d has an inhibitory effect on NLRP3, ASC, and Cleaved-Caspase1. Additionally, the experiment found that the ubiquitination level of NLRP3 significantly increased after SS-d treatment (*p* < 0.01). In summary, SS-d can improve the inflammatory state by promoting the ubiquitination of NLRP3 and inhibiting the activation of inflammasomes, thereby exhibiting anti-depressive effects on CUMS mice.

Yue Tong et al. [94] investigated the therapeutic effects of SS-a on depressed mice using the SPT, FST, and TST. The findings indicated that, following the administration of SS-a, there was an increase in the mice’s sucrose preference, as well as a reduction in the duration of immobility during FST and TST trials. These results suggest that SS-a exhibits antidepressant effects on CSDS mice. Further investigation into the effects of SS-a on hippocampal neurons was conducted. The results showed that, compared to the model group, the abnormal dendritic spine morphology in the hippocampal region of the mice in the administration group was improved, and the increase in the number of Ki67-positive nuclei in the dentate gyrus region of the hippocampus rose from 50.00% to 70.00%. Therefore, SS-a promotes hippocampal neurogenesis in mice subjected to CSDS and facilitates the repair of synaptic damage. Additionally, as the dose of SS-a increased, the increase in the Tet1/Tubulin ratio in the hippocampus of mice rose from 50.00% to 275.00%, the increase in the DLL3/Tubulin ratio rose from 66.67% to 150.00%, the increase in the Notch/Tubulin ratio rose from 100.00% to 220.00%, and the increase in the BDNF/Tubulin ratio rose from 75.00% to 275.00%. Meanwhile, Western blot analysis demonstrated that the knockdown of Tet1 reversed the SS-a-induced increase in DLL3, Notch, and BDNF expression in the hippocampal tissue of mice. In conclusion, SS-a promotes hippocampal neurogenesis by activating the Tet1/DLL3/Notch signaling pathway, thereby exerting an antidepressant effect in the CSDS mouse.

To investigate the antidepressant effects of *Bupleurum* total saponin (TSS) on chronic corticosterone-induced depression in ICR and C57BL/6J male mice, Xiuping Sun et al. [91] performed EPMs, NSFTs, and FSTs on mice that had been induced with chronic corticosterone. The findings indicate that following TSS administration, both the time spent in and the entry rate to the open arms of the maze increased during the EPM test (F(3,30) = 2.913, *p* < 0.05; F(3,30) = 3.527, *p* < 0.01). In the NSFT, the feeding latency of the mice was significantly reduced (F(3,30)= 6.927, *p* < 0.01) and in the FST, the duration of immobility decreased (F(3,37) = 7.512, *p* < 0.01). Therefore, TSS can ameliorate depression-like behavior in mice induced by chronic corticosterone. Western blot analysis was further employed to examine the impact of TSS on the AMPA receptor and mTOR signaling pathways in the hippocampus of mice subjected to chronic corticosterone. The results indicated that after TSS administration, the ratio of p-GluR1/GluR1 in the hippocampus of mice induced by chronic corticosterone increased by 350.00%, the ratio of p-mTOR/mTOR increased by 6.25%, the ratio of p-ERK/ERK increased by 33.33%, and the ratio of p-Akt/Akt increased by 200.00%. In summary, TSS improves depression-like behavior in chronic corticosterone-induced mice by enhancing AMPA receptor activity and activating mTOR signaling pathways. Consequently, TSS may serve as an effective therapeutic agent for depression.

As a crucial component of the neuroendocrine system, the hypothalamus–pituitary–adrenal axis (HPA) is considered to be a major contributor to depression [97,98]. Li et al. [93] administered SS-d treatment to rats subjected to CUMS. The results indicated that, following SS-d administration, sucrose consumption in the SPT increased and the movement distance in OFT increased (*p* < 0.05). Meanwhile, in the FST, the duration of immobility in the rats was reduced (*p* < 0.05). Furthermore, the study found that with the increase in SS-d dosage, the reduction in corticosterone (CORT) levels in rat serum increased from 28.81% to 35.59%, and it also decreased the expression of glucocorticoid receptors and the CUMS-induced nuclear translocation phenomenon. Therefore, SS-d demonstrates a therapeutic effect on depressed rats.

In the realm of neuropsychiatry, *Bupleurum* has shown promising antidepressant effects. Multiple animal models of depression (including chronic stress-induced and corticosterone-induced models) report behavioral and biochemical improvements after *Bupleurum* treatment. Table 4 provides a synopsis of these antidepressant findings, outlining the models used and the underlying mechanisms—such as activation of the cAMP/PKA/CREB pathway, inhibition of NLRP3 inflammasome-mediated neuroinflammation, and enhancement of neurogenesis via Tet1–Notch–BDNF signaling. The main molecular targets affected by *Bupleurum* in these studies are highlighted, illustrating a multi-pathway approach to alleviating depression. In summary, *Bupleurum*, as a traditional Chinese medicine, has broad prospects in the treatment of depression. Through comprehensive in-depth research and exploration, *Bupleurum* is expected to offer a more effective and safer solution for the treatment of depression.

**Table 4:** Antidepressant effects of *Bupleurum*: key mechanisms and targets in depression models.

Effect	Adopted Model	Main Mechanisms	Main Targets	References
Antidepressant	1. CUMS rats (*Bupleurum* extract) 2. CUMS mice (SS-d, SS-a) 3. Corticosterone-treated mice (TSS).	*Bupleurum* extract elevates cAMP/PKA/CREB, lowers CORT and inflammatory cytokines. SS-d fosters NLRP3 ubiquitination, inhibits inflammasome activation and hippocampal NF-κB/MAPK, while SS-a promotes hippocampal neurogenesis via Tet1–DLL3–Notch–BDNF. TSS activates AMPAR–mTOR signaling, improving depressive-like behavior.	cAMP, PKA, CREB, NLRP3, NF-κB, BDNF, mTOR, AMPAR	[90,91,92,94]

### 4.4. Anti-Aging

Aging is a complex natural phenomenon [99] characterized by systemic chronic inflammation, accompanied by cellular senescence, immunosenescence, organ dysfunction, and age-related diseases [100,101]. Reactive oxygen species (ROS) generated during cellular respiration, including compounds like hydrogen peroxide (H_2_O_2_), hydroxyl radicals (HO•), and superoxide ions (O^2−^) [102], can hasten the aging process. Consequently, the development of new anti-aging drugs has become a prominent topic in research.

In research into natural medicines, polysaccharides and their derivatives extracted from *Bupleurum* [103,104] have demonstrated excellent anti-aging properties. These active components primarily ameliorate aging-related pathological symptoms by regulating key signaling pathways such as p53-p21, p16-pRb, and NF-κB.

Haibin Tong et al. [103] isolated a polysaccharide known as BCPS-1 from the plant *Bupleurum*. They subsequently chemically modified this polysaccharide to produce two sulfonated derivatives, designated S-BCP1-4 and S-BCP1-8. The experiment was conducted to evaluate the three polysaccharides of BCPS-1, S-BCP1-4 and S-BCP1-8 by the 1,1-diphenyl-2-picrylhydrazyl (DPPH) radical assay, superoxide radical assay, and hydroxyl radical assay. The results indicate that BCPS-1, S-BCP1-4, and S-BCP1-8 exhibit scavenging capabilities for free radicals. To further investigate whether S-BCP1-4 and S-BCP1-8 can inhibit the aging of lung endothelial cells (MLECs) in H_2_O_2_-induced mice, flow cytometry was employed to analyze the cell cycle of MLECs. To further investigate whether S-BCP1-4 and S-BCP1-8 can inhibit the aging of lung endothelial cells (MLECs) in H_2_O_2_-induced mice, flow cytometry was employed to analyze the cell cycle of MLECs. The findings revealed that after H_2_O_2_ administration, 84.4% of MLECs were in the G0/G1 phase, while 10.8% were in the S phase. In comparison, following the administration of S-BCP1-4 and S-BCP1-8, the proportion of MLECs in the G0/G1 phase decreased to 63.6% and 60.3%, respectively, whereas the percentage of cells in the S phase increased to 28.8% and 30.5%. These results suggest that S-BCP1-4 and S-BCP1-8 effectively mitigate cell cycle arrest induced by oxidative stress, facilitating the transition of cells into the S phase and consequently attenuating the aging process. In addition, the experiments using qRT-PCR technology revealed that S-BCP1-4 reduced the expression level of p53 mRNA by 53.33% and that of p16^INK4a^ mRNA by 34.00%, while S-BCP1-8 decreased the expression level of p53 mRNA by 60.00% and that of p16^INK4a^ mRNA by 44.00%. In summary, S-BCP1-4 and S-BCP1-8 can prevent cellular senescence by inhibiting the p53-p21 and p16-pRb pathways. Therefore, *Bupleurum* polysaccharide and its sulfonated derivatives offer new possibilities for the clinical treatment of oxidative stress and aging and are expected to be developed as novel drugs for such diseases.

Macrophages play a vital role in both innate and acquired immunity, as well as in the process of inflammatory aging [105]. Mengran Xu et al. [104] investigated the effects of *Bupleurum* polysaccharide (BCP) on the production of cytokines by macrophages, specifically IL-1α, IL-6, and TNF-α. The research results indicate that as the BCP dosage increases, the reduction in IL-1α expression in macrophages rises from 61.67% to 83.33%, the reduction in IL-6 expression increases from 58.57% to 74.29%, and the reduction in TNF-α expression grows from 25.60% to 28.00%. Further ROS staining and fluorescence intensity analysis of macrophages revealed that BCP could reduce the level of ROS in lipopolysaccharide (LPS)-stimulated macrophages by 58.33%. Moreover, detection by JC-1 staining kit found that BCP has the effect of stabilizing mitochondrial membrane potential in LPS-stimulated macrophages. To investigate the impact of BCP on the aging of LPS-induced macrophages, we examined its effects on signaling proteins, including SA-β-gal, SAHF, p53, p16, and p65/NF-κB. The results indicated that BCP reduced the expression of SA-β-gal and SAHF in LPS-induced macrophages, and decreased the expression levels of p53 by 46.15%, p-p53 by 32.39%, p16 by 27.40%, and p-p16 by 33.33%. Concurrently, after BCP administration, the expression of p-p65 in macrophages decreased by 29.41%, the expression of p-I-κBα decreased by 40.00%, the expression of p65 decreased by 10.47%, and the expression of I-κBα increased by 60.00%. Therefore, BCP reduced the levels of inflammatory cytokines and the generation of oxidative stress by activating the NF-κB signaling pathway in LPS-stimulated macrophages, thereby hindering inflammatory aging.

*Bupleurum* also possesses anti-ageing and antioxidant effects at a cellular level. Table 5 compiles key findings from models of cellular senescence and oxidative stress, demonstrating how *Bupleurum* polysaccharides (and their chemically sulfated derivatives) mitigate aging processes. The table highlights mechanisms such as the prevention of cell cycle arrest (via down-regulation of p53/p21/p16 pathways) and reduction of reactive oxygen species (ROS) along with other senescence markers. In summary, *Bupleurum*, a traditional medicinal plant, holds significant promise in the domain of anti-aging. *Bupleurum* plays an active role in delaying aging and improving the health of the elderly through its anti-inflammatory, antioxidant, and immune-regulating properties. Nevertheless, further scientific evidence is required to comprehensively elucidate its mechanisms and potential applications, thereby offering new insights and strategies for the prevention and treatment of elderly-related diseases.

**Table 5:** Anti-ageing and antioxidant effects of *Bupleurum*: key mechanisms and targets in cellular models.

Effect	Adopted Model	Main Mechanisms	Main Targets	References
Anti-ageing/antioxidant	1. H_2_O_2_-induced senescent MLECs (S-BCP1-4, S-BCP1-8) 2. LPS-activated macrophages (BCP)	Sulfated BCP derivatives prevent G_0_/G_1_ arrest, down-regulate p53–p21 and p16–pRb pathways and favor S-phase entry. BCP lowers ROS, stabilizes mitochondrial membrane potential and represses NF-κB/p65, thereby reducing cellular senescence markers SA-β-gal and SAHF.	p53, p21, p16, NF-κB p65, ROS, SA-β-gal, SAHF	[103,104]

### 4.5. Anti-Epileptic

Epilepsy ranks as the second most common neurological disorder, impacting approximately 0.5–1% of the global population. It is characterized by higher drug resistance and poorer clinical outcomes compared to other neurological conditions [106]. Traditional Chinese medicine plays a significant role in the treatment of epilepsy through its multi-component, multi-pathway, and multi-target approach, exhibiting distinct characteristics and advantages. Therefore, it is crucial to investigate the nature of epilepsy and identify new targets for antiepileptic drugs (AEDs).

Traditional Chinese medicine demonstrates unique advantages in the treatment of epilepsy through its multi-component, multi-pathway, and multi-target approach. Compared with chemical drugs, traditional Chinese medicine demonstrates unique efficacy, fewer side effects, and a wealth of resources, garnering widespread attention both domestically and internationally. Research indicates that the flavonoids BCE-20 and BCE-70 [107] contained in *Bupleurum* can exert anti-epileptic effects by modulating the TREM/NF-κB/IκB signaling transduction pathway, while its volatile oil components [108] effectively ameliorate epilepsy-related symptoms through the regulation of dual signaling pathways involving Bcl2/Bax/caspase 3 and Notch1/GABA/GAD/GIRK.

Xiaomao Li et al. [107] extracted the flavonoid components BCE-20 and BCE-70 from *Bupleurum*. To investigate the anti-epileptic activity of the two components, a rat model of epilepsy induced by calcium (KA) was established in in vivo experiments, and in vitro experiments were used for LPS-induced BV2 microglia. The findings indicate that BCE-20 and BCE-70 significantly reduce the expression of IL-1β, IL-6, and TNF-α in KA-induced epilepsy rats, as well as inhibit the production of NO, IL-1β, IL-6, and TNF-α in LPS-induced BV2 microglia. Therefore, BCE-20 and BCE-70 can regulate neurotransmitter abnormalities and inhibit the expression and release of pro-inflammatory cytokines. REM2 plays a crucial role in neurodegenerative diseases and is the center of tissue damage signals, responding to tissue damage and undergoing immune remodeling. To investigate the mechanisms of action of BCE-20 and BCE-70 anti-epileptics, research demonstrated that both compounds reduced the phosphorylation levels of NF-κB and IκB in KA-induced epilepsy rats and LPS-induced BV2 microglia. Meanwhile, in LPS-induced BV2 microglial cells, BCE-20 and BCE-70 can increase the expression level of TREM2 protein and attenuate the activity of NF-κB. In summary, BCE-20 and BCE-70 inhibit neuroinflammation by inhibiting the TREM/NF-κB/IκB signaling pathway, thereby exhibiting anti-epileptic activity. Therefore, flavonoids of *Bupleurum* demonstrate a certain therapeutic effect on epilepsy.

To investigate the anti-epileptic properties of *Bupleurum* volatile oil (BAO), Xiaomao Li et al. [108] established a KA-induced epilepsy rat model for in vivo experiments. The findings indicated that following BAO treatment, there was an improvement in epilepsy symptoms, and the occurrence of status epilepticus (SE) was reduced (*p* < 0.05). To further elucidate the mechanism underlying the anti-epileptic action of BAO, hematoxylin and eosin (HE) staining and Nissl staining were employed. The results demonstrated that after BAO administration, the condition of pyramidal cells and Nissl bodies in the hippocampal CA3 region of mouse brain tissue improved. Concurrently, the phenomena of nuclear constriction, hypertrophy, astrocyte proliferation, and the disordered and irregular arrangement of pyramidal cell layers were also ameliorated. Immunohistochemical studies have indicated that BAO can improve the positive abnormal regions of rabbit-derived glial fibrillary acidic protein (GFAP) and activate astrocytes, thereby inhibiting the loss of hippocampal neurons. Neurotransmitters play a crucial role in the pathogenesis of epilepsy pain, with GABA [109] and glutamate serving as the primary transmitters involved in inhibition and excitation, and their metabolic abnormalities are closely related to the occurrence of epilepsy [110,111]. Furthermore, Western blotting was employed to measure the expression levels of relevant factors in rats. The study found that in KA-induced epileptic rats, BAO administration resulted in downregulation of Bcl-2 expression and upregulation of Bax and caspase-3 expressions. Concurrently, it reduced the expression levels of GAD65 by 38.89%, GAD67 by 36.84%, GIRK1 by 20.59%, and GFAP by 25.00%. This indicates that BA inhibits the Notch1/GABA/GAD/GIRK signaling pathway. In summary, BAO alleviates KA-induced epilepsy-like behaviors by modulating the Notch1/GABA/GAD/GIRK signaling pathway, thereby exerting a therapeutic effect on epilepsy.

This study revealed that following the administration of BAO, there was a decrease in the expression of Bcl-2, while the levels of Bax and caspase-3 were increased and the expression of GAD65, GAD67, GIRK1, and GFAP was inhibited. In summary, BAO mitigates KA-induced epilepsy-like behaviors by modulating the Bcl2/Bax/caspase-3 signaling pathway and the Notch1/GABA/GAD/GIRK signaling pathway, thereby playing a therapeutic role in epilepsy.

Studies have begun to explore *Bupleurum*’s anti-epileptic potential as part of its neuroprotective profile. Table 6 summarizes the evidence from seizure models, showing that *Bupleurum* extracts can attenuate epileptic symptoms and neuronal damage. Key mechanisms include the dampening of neuroinflammation (e.g., via NF-κB pathway inhibition) and the modulation of apoptosis and GABAergic signaling in the brain. The above studies indicate that *Bupleurum*, a traditional Chinese medicine, demonstrates significant anti-epileptic effects. With the backing of contemporary scientific advancements and ongoing in-depth investigations into the anti-epileptic mechanisms of *Bupleurum*, it has been shown to have important clinical value and extensive application prospects in the field of epilepsy treatment.

**Table 6:** Anti-Epileptic Effects of *Bupleurum*: Key Mechanisms and Targets in Epilepsy Models.

Effect	Adopted Model	Main Mechanisms	Main Targets	References
Antiepileptic	1. KA-induced rat epilepsy (BCE-20, BCE-70) and LPS-BV-2 cells 2. KA-induced rats treated with BAO	BCE-20/70 enhance TREM2, suppress NF-κB/IκB signaling and pro-inflammatory cytokines, reducing seizures. BAO modulates Bcl-2/Bax/caspase-3 and GABAergic pathways (GAD65/67, GIRK1), protecting hippocampal neurons.	TREM2, NF-κB, IL-1β/6, TNF-α, Bcl-2, Bax, caspase-3, GAD65/67, GIRK1	[107,108]

### 4.6. Antipyretic Effect

Fever is part of the body’s immune system, serving as an important response to disease and infection. *Bupleurum* can inhibit inflammatory exudation, granuloma formation, inflammation, capillary permeability, and release of inflammatory substances, thereby producing antipyretic effects. *Bupleurum* saponin has demonstrated efficacy in up to 95% of fever cases, particularly those induced by infections and the common cold.

Yuan Kang et al. [112] prepared *Bupleurum* water extract (BCE) and established a rat fever model induced by lipopolysaccharide (LPS) for in vivo experiments to investigate the antipyretic effect of BCE. The findings revealed that following the administration of BCE, the area under the curve (AUC) for rectal temperature was reduced compared to the model group, indicating that BCE effectively mitigates fever induced by LPS in rats. To study the antipyretic mechanism of BCE, LPS-induced complete blood cells (CBCs) were used for in vitro experiments. The results showed that BCE increased the reduction of TNF-α levels in the supernatant of CBCs from 31.03% to 42.53%. To further determine the effect of BCE on endogenous heat sources, the study involved in vivo experiments to establish a mouse endotoxinemia model. The results showed that the reduction in serum TNF-α levels in rats after BCE injection increased from 60.53% to 67.11%, while there was no effect on IL-6 and IL-1β. In conclusion, BCE exerts its antipyretic effects primarily by inhibiting TNF-α production in monocytes.

Table 7 outlines *Bupleurum*’s antipyretic effect in experimental fever models, noting the reduction in core body temperature in treated animals. The primary mechanism identified is the suppression of inflammatory mediators (notably TNF-α) that drive the fever response. This provides a scientific basis for *Bupleurum*’s efficacy in alleviating fever symptoms. In summary, *Bupleurum*, a traditional Chinese medicine, is widely utilized in clinical practice to alleviate fever resulting from various causes, and its antipyretic effect is superior to that of other traditional antipyretic drugs. This makes *Bupleurum* a promising natural antipyretic agent.

**Table 7:** Antipyretic effects of *Bupleurum*: key mechanisms and targets in fever models.

Effect	Adopted Model	Main Mechanisms	Main Targets	References
Antipyretic	LPS-induced fever rats and endotoxemic mice treated with BCE	BCE inhibits TNF-α release from peripheral monocytes, thereby lowering core body temperature in febrile animals.	TNF-α	[112]

### 4.7. Anti-Hepatitis C Virus

Hepatitis C virus (HCV) is classified as a positive-strand RNA virus belonging to the hepacivirus genus within the flaviviridae family [113]. It is the primary cause of chronic hepatitis, which can progress to cirrhosis and hepatocellular carcinoma [114,115]. Currently, direct antiviral agents (DAA) [116] have been developed that, while improving therapeutic outcomes, are expensive. Therefore, it is necessary to develop new and inexpensive anti-HCV methods.

Wei-Ping Lee et al. [117] conducted experimental research to verify the inhibitory impact of SS-b2 on HCV. Their results indicated that after 48 h of SS-b2 culture, luciferase activity in Huh-7.5.1 cells was reduced. Further research revealed that in Huh-7.5.1 cells, SS-b2 at a concentration of 100 µM reduced HCV translation by 37%, while it decreased HCV RNA replication by 55% and 77% for genotype 1b and genotype 2a, respectively. Moreover, experiments combining SS-b2 with Daclatasvir indicated that SS-b2 effectively inhibits Daclatasvir-resistant mutants, thereby preventing the resistance-associated substitutions (RAS) induced by Daclatasvir. In conclusion, SS-b2 exhibits therapeutic effects on HCV and may contribute to the development of a new combination therapy for this virus.

*Bupleurum* has also demonstrated antiviral activity, particularly against the hepatitis C virus (HCV). Table 8 highlights an in vitro study where *Bupleurum*’s saikosaponin B2 significantly inhibited HCV replication. In summary, *Bupleurum*, as a traditional Chinese medicine, exhibits significant potential for its antiviral effects. However, to fully validate the efficacy and safety of *Bupleurum* in clinical applications, further scientific evidence is necessary. In the future, *Bupleurum* is expected to assume an increasingly prominent role in the field of antiviral therapies.

**Table 8:** Antiviral effects of *Bupleurum* against HCV: key mechanisms and targets.

Effect	Adopted Model	Main Mechanisms	Main Targets	References
HCV	Huh-7.5.1 cells infected with HCV and treated with SS-b2	SS-b2 blocks HCV RNA translation and replication and suppresses Daclatasvir-resistant mutants, providing a potential combination therapy.	HCV RNA, NS5A pathway	[117]

### 4.8. Other Pharmacological Effects

#### 4.8.1. Anti-Alzheimer’s Disease

Alzheimer’s disease (AD) is a degenerative disorder of the central nervous system characterized by progressive dementia, which is accompanied by memory loss, cognitive impairment, and psychological deterioration [118]. The deposition of amyloid beta (Aβ) in the brain and blood vessels serves as the primary pathological marker of AD [119]. ZeHui Chen et al. [120] established an AD mouse model through the intraventricular injection of Aβ and discovered that *Bupleurum* saponin F exhibits significant therapeutic potential for AD. Additionally, ZhaoHan Huang et al. [121] employed molecular docking and network pharmacological methods to analyze the effects of *Bupleurum* and Paeonia lactiflora, finding that the combination can effectively treat AD.

#### 4.8.2. Anti-Cerebral Ischemic Injury

Cerebral ischemic injury is a common refractory disease that can result in significant and enduring disabilities [122]. Xinying Wang et al. [123] established a rat model of middle cerebral artery occlusion (MCAO) to evaluate cognitive and motor capabilities using behavioral testing. The results indicate that *Bupleurum* saponin A can alleviate brain damage in rats, promote the recovery of nerve function, reduce the moisture content in brain tissue, and exert a certain protective effect against cerebral ischemic injury.

#### 4.8.3. Anti-Allergic Asthma

Allergic asthma is an important global health problem and is a chronic disease characterized by symptoms such as wheezing, dyspnea, chest tightness, and cough [124,125,126]. The onset of symptoms typically occurs during the night or in the early morning hours. There is currently no specific drug and the main treatment strategy focuses on controlling symptoms. Consequently, there is an urgent need to develop safe and effective therapies for allergic asthma. Yandan Yin et al. [127] have demonstrated that *Bupleurum* saponin has a certain therapeutic effect on allergic asthma. Thi Tho Bui et al. [128] found through studies that extract of *Bupleurum* has the potential to treat allergic asthma.

#### 4.8.4. Anti-Cardiovascular Disease

Cardiovascular disease is one of the leading causes of death worldwide [129] and oxidative stress is an important pathogenesis of cardiovascular injury [130]. Junhui Gong et al. [131] discovered that *Bupleurum* polysaccharide protects cardiomyocytes from damage by inhibiting oxidative stress and mitochondria-mediated intrinsic apoptosis, thereby offering a potential treatment for cardiovascular diseases.

#### 4.8.5. Liver Protection

*Bupleurum* not only exhibits therapeutic properties for liver cancer but also offers protection for the liver and aids in the treatment of various hepatic disorders. Ya Zhao et al. [132] used the method of clamping the tail irritation to establish a rat model for liver depression and analyzed it through ultra-high performance liquid chromatography-tandem mass spectrometry (UPLC-MS/MS). The results indicated that *Bupleurum* has a protective impact on the liver, with SS-b2 recognized as a crucial active component. Yi Wang et al. [133] discovered that Xiao Chaihu Decoction has a certain therapeutic effect on hepatitis and liver fibrosis. Reneta Gevrenov et al. [134] found that the flavonoid compounds narcissus and rutin in *Bupleurum* can effectively inhibit liver damage caused by carbon tetrachloride (CCl4) and tert-Butyl hydroperoxide (t-BuOOH), thereby providing a protective effect on the liver. Kehui Zhang et al. [135] established a wild-type and ERKO mouse liver fibrosis model, demonstrating that SS-d can inhibit the ROS/NLRP3 inflammasome axis through the activation of the ERβ pathway, thereby alleviating liver fibrosis.

#### 4.8.6. Antidiabetics

Diabetes is a chronic condition resulting from inadequate insulin secretion or insulin resistance in peripheral blood tissues, characterized by hyperglycemia, lipid damage, and disorders in protein metabolism [136,137]. This disease can result in a range of serious complications, including cardiovascular disease, retinopathy, neuropathy, and kidney disease [138]. Currently, antidiabetic drugs, such as insulin, biguanides, sulfonylureas, and α-glucosidase inhibitors [139], can control blood sugar levels, but the treatment effect is average, and has many serious side effects [140]. Lingyu Pan et al. [141] utilized a diabetic mouse model induced by streptozotocin (STZ) to demonstrate that *Bupleurum* polysaccharide exhibits therapeutic effects on diabetes and its associated complications.

## 5. VOSviewer

In this study, we utilized VOSviewer (version 1.6.20), a software package specifically designed for bibliometric analysis and the creation of scientific knowledge maps, to generate a visual network diagram. This tool systematically presents research topics, academic activities, and literature sources related to *Bupleurum*, significantly enhancing the depth and structured approach of our literature review analysis.

Node: Each node represents a keyword extracted from the literature, with its size visually indicating the frequency or importance of the keyword in academic publications. The larger the node, the more extensively the keyword is discussed in *Bupleurum* research, indicating a more significant academic influence.

Edges: The lines between nodes represent the co-occurrence relationship of keywords, indicating the frequency at which two keywords appear together in the same document. The thicker or denser the line, the stronger the association of these keywords in the research.

Cluster colors: Through algorithmic analysis, VOSviewer automatically groups closely related keywords into the same cluster and labels them with the same color. For example, all red nodes may represent studies on the “pharmacological activity” of *Bupleurum*, while blue nodes may focus on “cultivation techniques.” This classification reveals the intrinsic connections between different research directions, and keywords within the same cluster are more likely to co-occur in the literature.

Through this visual analysis, we are able to quickly identify the core themes, emerging trends, and interdisciplinary intersections in the field of *Bupleurum* research, thereby providing data support for subsequent studies (Figure 5).

## 6. Conclusions

*Bupleurum*, as a traditional Chinese medicine with a long history of application, has seen its extensive therapeutic potential widely validated in modern pharmacological research. Bibliometric analysis based on VOSviewer reveals that the current research field of *Bupleurum* focuses on two core directions: the material basis of pharmacodynamics and multi-dimensional pharmacological mechanisms. On one hand, studies have deeply analyzed the structures of active chemical components represented by SS-d, SS-a, and SS-b2. On the other hand, extensive exploration has been conducted on the mechanisms of *Bupleurum* and its active components in anti-tumor, anti-inflammatory, neuroprotective, antiviral, and antipyretic effects. The high-frequency keyword clusters in the mapping analysis, such as “Saikosaponin d”, “anti-tumor”, “NF-κB”, “inflammation”, “depression”, and “polysaccharides”, precisely correspond to the key chemical components, primary indications, and in-depth molecular mechanisms discussed in the main text. This reflects the current research paradigm of the “chemical-activity-mechanism” trinity in the study of *Bupleurum*, with a particular emphasis on employing modern molecular biology techniques to elucidate the scientific connotation of its traditional efficacy, thereby laying a solid knowledge foundation for subsequent in-depth development.

Moreover, *Bupleurum* demonstrates unique values distinct from single-component Western medicines. Its multi-component characteristics enable it to simultaneously influence multiple aspects of disease networks. For instance, in anti-tumor applications, it can induce apoptosis, inhibit proliferation, and exert anti-inflammatory effects; in anti-depression, it concurrently regulates neurotransmitters, inhibits neuroinflammation, and promotes neurogenesis, potentially offering more comprehensive efficacy against complex diseases. Secondly, *Bupleurum*’s long history of application in traditional Chinese medicine clinical practice provides a valuable experiential foundation for modern drug development. Finally, as a natural plant source, it possesses relatively renewable characteristics, aligning with the needs of sustainable development. To fully explore and utilize the potential of *Bupleurum*, future research needs to delve deeper into elucidating the mechanisms of action of its multiple components, particularly by strengthening the study of multi-component effects based on network pharmacology on the foundation of deepening existing understanding. Simultaneously, it is essential to accelerate the advancement of high-quality clinical translation, confirming its efficacy and safety through rigorously designed clinical trials to clarify its unique value in the modern healthcare system.

## 7. Challenges and Breakthrough Directions

In recent years, with the sustained development of the national economy, people’s demands for quality of life and medical treatment have been continuously increasing. A large number of studies have confirmed that *Bupleurum*, as a natural Chinese herbal medicine, possesses extensive pharmacological effects, but its clinical application still faces numerous challenges. The main active components such as saikosaponins face the issue of low oral bioavailability [142]. However, their complex and variable pharmacokinetic processes in vivo, along with the current lack of systematic and reliable human pharmacokinetic data, make it difficult to optimize clinical dosing regimens and achieve personalized medication. Meanwhile, the safety evaluation system for *Bupleurum* and its preparations urgently needs improvement. Known adverse effects include allergic reactions, gastrointestinal discomfort, and the risk of hepatotoxicity from long-term or high-dose use. Of particular concern are its potential drug interaction risks. When used in combination with Western medicines, *Bupleurum* can affect drug absorption or efficacy due to chemical reactions when used with metal ion drugs such as Compound Aluminum Hydroxide Tablets and Ferrous Gluconate Tablets. It increases the risk of bleeding when combined with anticoagulants. In traditional Chinese medicine formulations, although *Bupleurum* can be paired with various herbs like *Scutellaria baicalensis* and *Paeonia lactiflora* to enhance efficacy, there are still some adverse reactions. The concurrent use with dietary supplements such as grapefruit juice, certain vitamins, or mineral supplements may affect drug-metabolizing enzymes or transporters. Currently, there is a severe lack of systematic mechanism research and clinical risk assessment for these complex interactions. Additionally, variations in the sources of medicinal materials and changes in composition due to non-standardized processing techniques may also significantly influence their toxic manifestations. Therefore, strengthening quality control is a fundamental step in ensuring safety. For special populations such as the elderly, individuals with impaired liver or kidney function, children, pregnant women, and lactating women, the sensitivity and safety data of *Bupleurum* remain severely lacking. There is an urgent need for targeted research to assess its potential risks in different physiological and pathological conditions.

In summary, future research should focus on in-depth analysis of the pharmacokinetic properties of *Bupleurum*, clarifying its toxicity mechanisms and the patterns of drug interactions, systematically filling the gaps in safety data for special populations, and exploring and evaluating the advantages, limitations, and translational feasibility of novel delivery systems, including nanotechnology, in enhancing bioavailability, achieving targeted drug delivery, and reducing systemic toxicity. By systematically and multidimensionally overcoming these core scientific bottlenecks, robust evidence-based foundations can be established, ultimately providing more support for the safe, effective, and precise application of *Bupleurum* and its active components in modern clinical practice.

## Data Availability

No new data were created or analyzed in this study.
